# Valence-skipping and quasi-two-dimensionality of superconductivity in a van der Waals insulator

**DOI:** 10.1038/s41467-022-34726-3

**Published:** 2022-11-14

**Authors:** Caorong Zhang, Junwei Huang, Kun Zhai, Keivan Akhtari, Zhiwei Shen, Lingyi Ao, Zeya Li, Feng Qin, Yukai Chang, Ling Zhou, Ming Tang, Xueting Dai, Caiyu Qiu, Yi Zhang, Lin Wang, Zhongyuan Liu, Yongjun Tian, Mohammad Saeed Bahramy, Hongtao Yuan

**Affiliations:** 1 National Laboratory of Solid State Microstructures & College of Engineering and Applied Sciences, Nanjing University, Nanjing, 210093 China; 2 School of Physics, Nanjing University, Nanjing, 210093 China; 3 Collaborative Innovation Center of Advanced Microstructures, Nanjing University, Nanjing, 210093 China; 4 Jiangsu Key Laboratory of Artificial Functional Materials, Nanjing University, Nanjing, 210093 China; 5 Center for High Pressure Science, Yanshan University, Qinhuangdao, 066004 China; 6 State Key Laboratory of Metastable Materials Science & Technology, Yanshan University, Qinhuangdao, 066004 China; 7 Department of Physics, University of Kurdistan, Sanandaj, 416 Iran; 8 Department of Physics and Astronomy, The University of Manchester, Oxford Road, Manchester, M13 9PL UK; 9 School of Natural Sciences, The University of Manchester, Oxford Road, Manchester, M13 9PL UK

**Keywords:** Phase transitions and critical phenomena, Superconducting properties and materials, Two-dimensional materials, Electronic structure

## Abstract

Valence fluctuation of interacting electrons plays a crucial role in emergent quantum phenomena in correlated electron systems. The theoretical rationale is that this effect can drive a band insulator into a superconductor through charge redistribution around the Fermi level. However, the root cause of such a fluctuating leap in the ionic valency remains elusive. Here, we demonstrate a valence-skipping-driven insulator-to-superconductor transition and realize quasi-two-dimensional superconductivity in a van der Waals insulator GeP under pressure. This is shown to result from valence skipping of the Ge cation, altering its average valency from 3+ to 4+, turning GeP from a layered compound to a three-dimensional covalent system with superconducting critical temperature reaching its maximum of 10 K. Such a valence-skipping-induced superconductivity with a quasi-two-dimensional nature in thin samples, showing a Berezinskii-Kosterlitz-Thouless-like character, is further confirmed by angle-dependent upper-critical-field measurements. These findings provide a model system to examine competing order parameters in valence-skipping systems.

## Abstract (web summary)

Fluctuation of the cation valence state plays a crucial role in emergent quantum phenomena in correlated electron systems. Here, the authors demonstrate a valence-fluctuation-driven insulator-to-superconductor transition in the van der Waals insulator GeP as a function of hydrostatic pressure.

## Introduction

Valence skipping is a novel quantum phenomenon that can profoundly affect the collective behavior of electrons in a solid^1–3^. Variation in the valent state of the host cations enables a material to exhibit a wide variety of physical and chemical phases, ranging from insulator, metal, to superconductor. Notable examples are the valence-skipping-induced superconductivity observed in Ba_1–*x*_K_*x*_BiO_3_ compounds^1–4^ and Te-based chalcogenide alloys^5,6^, in which the valent state of those cations in a lattice can appear in two or more characteristically different variants. The underlying mechanism controlling the cationic valent state can be phenomenologically described by the Hubbard model with negative*-U* interactions^7–9^. Within this scheme, the electronic short-range Coulomb interactions couple electrons in pairs in real space, usually giving rise to an insulating phase. A longstanding question has been whether such localized electron pairs can also melt into a Fermi liquid with a superconducting ground state once the cationic valent state is altered^5,10–13^. While extensive theoretical studies have attempted to find an answer to this question^14–17^, less has been done experimentally, mainly owing to the practical challenges hindering any systematic control on the valent state, especially if the system is subject to strong correlation effects.

Here, we demonstrate an insulator-to-superconductor transition through a peculiar valence-skipping mechanism in a bulk van der Waals insulator GeP, with the combination of diamond-anvil-cell (DAC) high-pressure measurements and first-principles calculations. We observe a phase transition, starting from an initial insulating state to an emergent superconducting state at a quantum critical pressure ${P}_{{{{{{\rm{QCP}}}}}}}$ of ~15.0 GPa, followed by a dome-like-shaped superconducting phase diagram with a maximum critical temperature ${T}_{{{{{{\rm{c}}}}}}}$ of 10.0 K at ~23.0 GPa. Using first-principles calculations, we find that after the band gap closes at ${P}_{{{{{{\rm{QCP}}}}}}}$, the valence skipping at the Ge site (altering its cationic state from the averaged 3+ to 4+ valence state at a pressure of ~23.0 GPa) can cause a structural phase transition and further turn GeP from a van der Waals layered compound to a three-dimensional covalent system. These two phase transitions are indicated by the threshold point and tipping point of the superconducting phase diagram, the latter is consistent with the confirmations of high-pressure X-ray diffraction (XRD) and Raman spectra. Remarkably, the quasi**-**two-dimensional (quasi**-**2D) nature of the observed superconductivity has been confirmed in pressurized GeP thin flakes based on the 2D Tinkham analysis for the angle-dependent upper critical field and the Berezinskii–Kosterlitz–Thouless (BKT) analysis. These findings suggest that GeP can serve as an ideal platform for investigating valence-skipping-related quantum effects in low-dimensional correlated electronic systems and exploring their possible functionalities under controlled conditions.

## Results

### Valence-skipping-driven insulator-superconductor transition

**Fig. 1:**
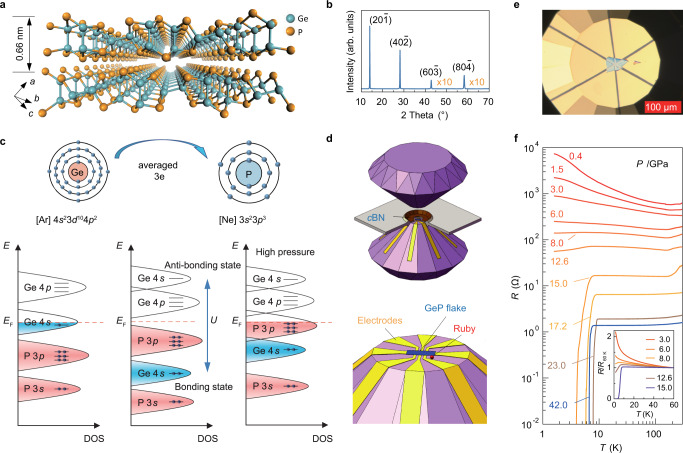
Crystal structure, electron filling in orbitals, and insulator–superconductor transition of valence-skipping GeP van der Waals crystal under pressure. **a** Schematic crystal structure of GeP. Cyan and orange balls represent Ge and P atoms. **b** XRD spectrum of GeP single crystal. **c** The electron configuration of Ge and P atoms. Bottom panels are schematic illustrations of the DOS in different scenarios for electron filling states. GeP should be a metal with a half-filled Ge-4*s* orbital according to the band theory^18^ (left panel), however, it is an insulator realized by the negative-*U* interaction between electrons splitting the 4*s* orbital (middle panel), forming a mixed valent state GeP. The band gap closes under pressure (right panel). **d** Schematics for the high-pressure DAC setup with a transferred GeP flake. **e** Optical microscope image of GeP thin flake and pre-patterned Ti/Au electrodes on the diamond culet. The scale bar is 100 μm. **f** Temperature-dependent four-terminal resistance of GeP (sample 1) under various pressures. The inset shows the normalized resistance $R/{R}_{60{{{\,{{\rm{K}}}}}}}$ (${R}_{60{{{\,{{\rm{K}}}}}}}$ is the resistance at 60 K) from 3.0 to 15.0 GPa as a function of temperature.

As schematically shown in Fig. 1a, GeP is a layered van der Waals compound, crystalizing in a monoclinic structure (space group of $C2/m$, verified by powder XRD results shown in Supplementary Fig. 1) with a preferential lattice orientation $[20\bar{1}]$, as confirmed by our single-crystal XRD measurements (Fig. 1b) and atomic-scale high-angle annular dark-field image of scanning transmission electron microscopy for the GeP crystal (Supplementary Fig. 2). Under ambient conditions, GeP behaves like an insulator with the Fermi level falling into a band gap, as shown in the schematic band structure in Fig. 1c. This is attributed to the unusual valence state of Ge cations in GeP. By default, Ge prefers a Ge^2+^ cationic state, and P takes a P^3−^ anionic state. However, this combination is imbalanced by one electron. Due to the strong electronegativity of P, Ge is thus pushed to an averaged 3+ state (2+ and 4+ admixture) to balance the negative charge of P^3−^ and form neutral GeP. Since the electron configuration of Ge atoms is [Ar]4*s*^2^3*d*^10^4*p*^2^, the Ge^3+^ valence state will make its 4*p* orbital empty while leaving one electron in its 4*s* shell^18,19^. However, such a partial occupation is not stable enough to resist strong electron interactions in two dimensions and thus undergoes a metal-insulator phase transition. To accommodate this, Ge-4*s* is split into bonding and anti-bonding states^20,21^, appearing below and above the Fermi level, thereby providing the extra electron to P to form the band gap. Specifically, each Ge atom is covalently surrounded by three P atoms and one Ge atom, in which three Ge−P covalent bonds are fully occupied while the Ge−Ge covalent bond is half-filled. The external pressure enables tuning of this valence mixture and its insulating phase. This is theoretically expected to lower the repulsive Coulomb interactions between the electrons at the Fermi level by redistributing them in new bonding channels. As a result, the band gap vanishes at a quantum critical pressure, opening the way for the emergence of superconductivity.

**Fig. 2:**
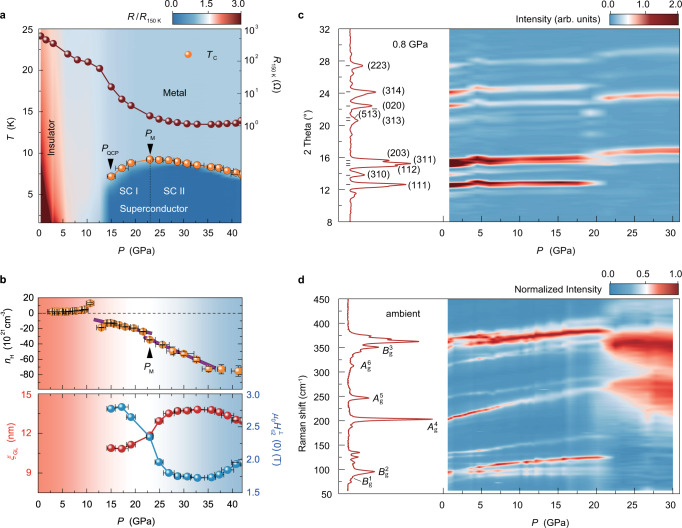
Phase diagram of the superconductivity of GeP and its structural phase transition under pressure. **a** Color mapping of the normalized resistance $R/{R}_{150{{{\,{{\rm{K}}}}}}}$ as a function of temperature and pressure for GeP (sample 1). The insulating phase is shaded in red, and the superconducting phases (SC I and SC II) are shaded in blue. The orange balls represent the superconducting critical temperature ${T}_{{{{{{\rm{c}}}}}}}$, which is defined as the temperature at which resistance drops to 90% of the$\,{R}_{{{{{{\rm{N}}}}}}}$ (${R}_{{{{{{\rm{N}}}}}}}$ denotes the resistance of the normal state at 14 K). The pressure corresponding to the insulator-superconductor transition is denoted as the quantum critical pressure ${P}_{{{{{{\rm{QCP}}}}}}}$. ${P}_{{{{{{\rm{M}}}}}}}$ is defined by the critical pressure where the structural phase transition occurs with maximum ${T}_{{{{{{\rm{c}}}}}}}$ and can be used to distinguish SC I and SC II phases. The dark red balls denote the resistance values at 150 K. The error bars on pressure are determined by the differences in fluorescence lines at different positions on the same ruby. **b** Top panel presents the carrier density as a function of pressure for sample 2 at 1.5 K. The carrier density is deduced from the Hall effect measured at 1.5 K by applying magnetic fields to ±8 T (larger than the ${H}_{{{{{{\rm{c}}}}}}2}$, which is defined as the magnetic field at which resistance drops to 90% of the$\,{R}_{{{{{{\rm{N}}}}}}}$). The purple guidelines are added to emphasize a jump in carrier density values at around ${P}_{{{{{{\rm{M}}}}}}}$. The error bars on carrier density represent the uncertainty arising from determining the linear regime for the fit. The bottom panel shows the pressure-dependent ${H}_{{{{{{\rm{c}}}}}}2}^{\perp }\left(0\right)$ (blue balls) and ${\xi }_{{{{{{\rm{GL}}}}}}}\left(0\right)$ (red balls) of sample 1. The error bars on pressure are determined by the differences in fluorescence lines at different positions on the same ruby. The red shaded area corresponds to quasi-2D monoclini**c** structure, and the blue-shaded area corresponds to bulk monoclinic structure. **c** Color mapping of XRD patterns plotted as a function of pressure and diffraction angle at room temperature. The left panel shows the XRD spectrum at 0.8 GPa, and the main diffraction indexes are labeled. The wavelength of the X-ray is *λ* = 0.7107 Å. **d** Color mapping of normalized Raman spectra plotted as a function of pressure and Raman vibration frequency at room temperature. The left panel shows an individual Raman spectrum at ambient pressure.

To experimentally observe such a superconducting phase, we employed the DAC technique (Fig. 1d) to apply a fine-tuned high pressure up to 50.0 GPa for electronic transport measurements (the optical image of the GeP flake-based device is shown in Fig. 1e). Figure 1f shows the temperature-dependent electrical resistance $R(T)$ in a bulk-like GeP (sample 1) at various pressures (more results with different thicknesses are shown in Supplementary Fig. 3). The observed $R\left(T\right)$ curves exhibit an insulator-superconductor phase transition. As shown in the inset of Fig. 1f, GeP turns from an insulator to a superconductor, accompanied by the closing up of the thermal activation gap (Supplementary Fig. 4) when the pressure reaches ${P}_{{{{{{\rm{QCP}}}}}}}$ of ~15.0 GPa, appearing as a zero-resistance superconducting state below 4.0 K. Figure 2a shows the corresponding ${T}_{{{{{{\rm{c}}}}}}}\left(P\right)$ phase diagram of GeP (sample 1). It clearly shows distinct pressure-temperature regions in which the system behaves as the insulator (shaded in red), the metal (shaded in pale blue), and the superconductor (shaded in dark blue). Interestingly, the region of superconductivity is a dome-like shape, with a maximum ${T}_{{{{{{\rm{c}}}}}}}$ of ~10.0 K at the pressure ${P}_{{{{{{\rm{M}}}}}}}$ ~23.0 GPa. Note that we define ${P}_{{{{{{\rm{M}}}}}}}$ based on the identification of the structural phase transition in GeP (evidence will be provided later in Fig. 2c, d), in which two different superconducting phases, namely SC I and SC II, are separated by ${P}_{{{{{{\rm{M}}}}}}}$: the SC I phase corresponds to the layered monoclinic structure with the $C2/m$ space group, while the SC II phase corresponds to the three-dimensionally coordinated monoclinic structure. In Fig. 2a, we also show the pressure dependence of resistance at 150 K (${R}_{150{{{\,{{\rm{K}}}}}}}$). As can be seen, it monotonically decreases with increasing pressure. At 12.6 GPa near ${P}_{{{{{{\rm{QCP}}}}}}}$, a kink feature appears in the ${R}_{150{{{\,{{\rm{K}}}}}}}(P)$ curve corresponding to the close-up of the band gap. Upon further increasing the pressure, the ${R}_{150{{{\,{{\rm{K}}}}}}}$ values show a smooth trend to saturation and are less sensitive to pressure, indicating that the GeP evolves into the metallic state.

To further understand the tuned electronic states of the dome-like-shaped superconductivity, we performed transport measurements for the Hall coefficient (Supplementary Figs. 5 and 6) and superconducting coherence length (Supplementary Figs. 7 and 8). Interestingly, the Hall coefficient shows a sign change from positive to negative values at ${P}_{{{{{{\rm{QCP}}}}}}}$, and the major carrier type changes from hole to electron (top panel of Fig. 2b, sample 2), providing insight into the electronic state change in the band structure at a pressure where the zero-resistance superconducting state appears. In the electron-dominating region at high pressure, there is a clear jump in carrier density values at ${P}_{{{{{{\rm{M}}}}}}}$, corresponding to another electronic state change at this pressure. We further deduced the in-plane Ginzburg–Landau (GL) coherence length ${\xi }_{{{{{{\rm{GL}}}}}}}\left(0\right)$ of the SC I and SC II phases from the out-of-plane upper critical field ${H}_{{{{{{\rm{c}}}}}}2}^{\perp }\left(T\right)$ based on the GL formula,

1 $${\mu }_{0}{H}_{{{{{{\rm{c}}}}}}2}^{\perp }\left(T\right)=\frac{{\phi }_{0}}{2\pi {{\xi }_{{{{{{\rm{GL}}}}}}}\left(0\right)}^{2}}\left(1-\frac{T}{{T}_{{{{{{\rm{c}}}}}}}}\right)$$

where$\,{\phi }_{0}$ is the magnetic flux quantum. As shown in the bottom panel of Fig. 2b, the ${H}_{{{{{{\rm{c}}}}}}2}^{\perp }\left(0\right)$ values start to drop from 2.8 T (at 15.0 GPa) to a saturated value around 1.8 T (at 27.0 GPa), which is almost independent of the further increasing pressure. Correspondingly, the enhancement of ${\xi }_{{{{{{\rm{GL}}}}}}}\left(0\right)$ values from 10.8 nm (at 15.0 GPa) to 13.5 nm (at 27.0 GPa) can be achieved, indicating that the spatial extension of superconducting electrons is enhanced in the high-pressure structure of GeP. Note that the coherence length slightly decreases when the pressure is above 35.0 GPa, which might be related to the decreasing trend of ${T}_{{{{{{\rm{c}}}}}}}$ itself. Such two-step-like behavior directly suggests the variation of electron pairing in SC I and SC II phases, which are associated with two different crystal structures (to be discussed later).

### Structural phase transition with valence state skipping

To gain insight into the structural phase transition of GeP under pressure, we performed XRD and Raman spectroscopy measurements. As shown in Fig. 2c, at low pressures, all Bragg diffraction peaks in the XRD spectra can be well indexed by a monoclinic crystal structure with the ${C}2/m$ space group (more details shown in Supplementary Figs. 9 and 10). With increasing pressure, the Bragg peaks shift to larger angles (right panel in Fig. 2c), indicating the lattice parameters are continuously compressed under pressure. The Bragg peaks of $\left(111\right)$, $\left(313\right)$, and $\left(314\right)$ are strongly suppressed at ${P}_{{{{{{\rm{M}}}}}}}$, while the Bragg peaks of $\left(020\right)$ and$\,\left(223\right)$ remain and do not become broad when the pressure is higher than ${P}_{{{{{{\rm{M}}}}}}}$, indicating a structural phase transition at this pressure. Such a critical pressure value is consistent with that for the electronic transition between SC I and SC II phases shown in Fig. 2a, b. The discussion of the observed Raman spectra is focused on the following main active phonon vibrations at ambient pressure: the ${B}_{{{{{{\rm{g}}}}}}}^{1}$ mode around 79 cm^–1^, the ${B}_{{{{{{\rm{g}}}}}}}^{2}$ mode around 96 cm^–1^, the ${A}_{{{{{{\rm{g}}}}}}}^{4}$ mode around 203 cm^–1^, and the ${A}_{{{{{{\rm{g}}}}}}}^{6}$ mode around 312 cm^–1^ (Fig. 2d, left panel). The mapping data in Fig. 2d show the evolution of these phonon modes with pressure (more details are given in Supplementary Figs. 11 and 12). One can see that the ${B}_{{{{{{\rm{g}}}}}}}^{1}$ and ${B}_{{{{{{\rm{g}}}}}}}^{2}$ modes around 79 and 96 cm^–1^ show a crossover at a relatively low pressure of ~1.5 GPa (Supplementary Fig. 11) and disappear at ${P}_{{{{{{\rm{M}}}}}}}$, whereas the other two peaks ${A}_{{{{{{\rm{g}}}}}}}^{4}$ and ${A}_{{{{{{\rm{g}}}}}}}^{6}$ display a clear blueshift at first and later evolve into two broad peaks around 275 cm^–1^ and 362 cm^–1^ above ${P}_{{{{{{\rm{M}}}}}}}$. Such a dramatic change in the Raman spectra is reversible before compression and after decompression (more details shown in Supplementary Fig. 12), clearly ruling out the amorphization of the GeP crystal under high pressure and providing direct evidence for the occurrence of the structural phase transition at ${P}_{{{{{{\rm{M}}}}}}}$ (associated with the valence-skipping mechanism). From 30.0 GPa, the intensity of all Raman peaks decreases quickly to the background noise level with increased pressure. Such signal weakening is likely due to the great enhancement of the electronic density of states (DOS) near the Fermi level, making the surface of the pressurized metallic sample too reflective to generate a Raman signal.

### Electronic structure and valence state evolution

**Fig. 3:**
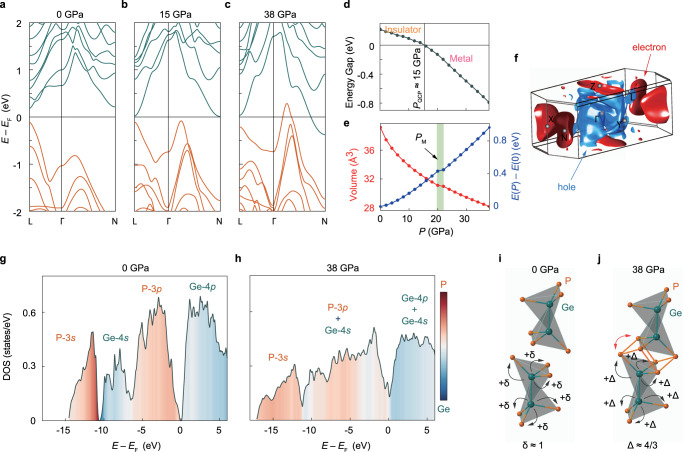
Electronic structure and valence-skipping evolution of GeP under pressure. The electronic band structure at **a** ambient pressure, **b** 15.0 GPa and **c** 38.0 GPa. **d** The pressure dependence of the energy band gap. One can see a clear insulator-to-metal (superconductor) phase transition at the quantum critical pressure ${P}_{{{{{{\rm{QCP}}}}}}} \sim 15.0$ GPa. **e** Differential free energy $E\left(P\right)-E(0)$ (blue curve) and unit-cell volume per formula unit (red curve). The green shaded area corresponds to the pressure range within which a structural phase transition takes place at critical pressure ${P}_{{{{{{\rm{M}}}}}}}$ based on the valence-skipping mechanism. **f** The corresponding Fermi surface of GeP at 38.0 GPa. The hole and electron pockets are shown in blue and red, respectively. The labels denote the high symmetry *k*-points in the BZ. The corresponding orbital-projected density of states at **g** ambient pressure and **h** 38.0 GPa. Schematic illustration of the pressure-induced structural phase transition in GeP from **i** a quasi-2D monoclinic structure to **j** a bulk monoclinic structure. $\delta$ and $\varDelta$ denote the charge transferred from Ge to each P at 0 and 38.0 GPa, respectively.

To understand how the applied pressure tunes the electronic and structural properties, as well as valence state of bulk-like GeP, we performed first-principles calculations considering hydrostatic pressures up to 40.0 GPa. We have fully optimized the lattice parameters and atomic position at each pressure to account for any possible structural phase transition within this range. As shown in Fig. 3, these calculations reveal remarkable changes in both the electronic and volumetric properties of GeP under pressure. The most evident change is the insulator-metal phase transition at a quantum critical pressure of 15.0 GPa (Fig. 3a, b). At ambient pressure, the system clearly has a narrow direct band gap appearing at one of the side centers (L point) of its Brillouin zone (BZ). This gap separates the predominantly P-3*p*-type valence bands from the Ge-4*p* conduction bands. By applying pressure, the lowest conduction band (dominated by Ge-4*p* orbitals) at and in the vicinity of the BZ’s side center N, is pushed downwards. Concurrently, the topmost valence band forms a hump along the Γ–N direction. This contrasting behavior eventually leads to an indirect band gap closing at 15.0 GPa (Fig. 3d) and causes a noteworthy change in the type of major charge carriers from hole to electron (Fig. 2b). However, the band gap closing does not occur along the high symmetry directions in the BZ. As seen from the Fermi surface at 18.0 GPa in Supplementary Fig. 13a, the hole pockets emerge at some generic *k*-points within the central region of the BZ. The electron pockets are localized around the Zone centers, N point, making the whole system behave as a compensating semimetal with an indirect overlap between the electron and hole pockets. This pressure of 15.0 GPa excellently matches the ${P}_{{{{{{\rm{QCP}}}}}}}$ value in our transport experiment. As such, we expect the observed superconductivity (SC I) to emerge from such a compensated semi-metallic phase with electron and hole pockets, filling different parts of the BZ.

Upon further increasing the pressure, we continue to see the same trend of band shifting between the conduction and valence bands (Fig. 3c). Moreover, as shown in Fig. 3d, the rate of change becomes significantly steeper for $P > {P}_{{{{{{\rm{QCP}}}}}}}$, suggesting new charge transfer pathways between Ge and P associated with valence fluctuation. As such pathways usually tend to re-coordinate the ions, they can potentially promote structural phase transition in the whole system. To verify this, we have calculated the pressure dependence of the free energy $E$ and lattice volume $V$ (Fig. 3e). Astonishingly, these calculations reveal an apparent step-like anomaly in both $E\left(P\right)$ and $V(P)$ between 20.0 GPa and 22.0 GPa, again in perfect agreement with the experimentally observed ${P}_{{{{{{\rm{M}}}}}}}\approx$ 23.0 GPa (Fig. 2). This result accordingly confirms that GeP is structurally modified under hydrostatic pressures at and beyond ${P}_{{{{{{\rm{M}}}}}}}$, becoming a bulk-like monoclinic structure. The structure modification can also be substantiated by comparing the calculated XRD results with those experimental observations (Fig. 2c). Within the superconducting regime SC II of the phase diagram, the BZ is massively occupied by cylindrical hole pockets at the center surrounded by three-dimensionally dispersed electron pockets (taking 38.0 GPa as an example in Fig. 3f, more results in Supplementary Fig. 13). They should accordingly allow a large Fermi surface nesting, which in turn could facilitate Cooper pairing in the respective SC II phase. Their vast coverage of the BZ can also explain why the measured resistance shows less sensitivity to pressure above ${P}_{{{{{{\rm{M}}}}}}}$ (Fig. 2a).

Figure 3g, h compares the DOS calculated at ambient pressure and 38.0 GPa, before and after the pressure-induced valence skipping. The differences are apparent. At ambient pressure, there is a clear separation between the Ge and P states. Above the Fermi level, Ge-4*p* orbitals dominate the DOS, whereas, at lower energies, the P-3*p* and P-3*s* orbitals give a major contribution. In a small energy window (–10 eV to –5 eV) below the Fermi level, Ge-4*s* orbitals form a distinct bonding state between the P-3*p* and P-3*s* orbitals. Within this energy window, each Ge shares one electron with its adjacent Ge to form Ge-Ge bridges within the GeP layer, and splits the Ge-4*s* band into two sub-bands below and above the Fermi level. The remaining Ge-4*s* electron is then hybridized with P-3*p*. This Ge-4*s* electron, in addition to two electrons taken from Ge-4*p* orbitals, enforces the Ge^3+^ state needed for the insulating phase of GeP. At 38.0 GPa, the band gap has completely vanished, and there is no trace of Ge-4*s* bands below the Fermi level. They have now been either hybridized with P-3*p* bands or shifted above the Fermi level. As such, Ge is now expected to be in the Ge^4+^ valence state. The additional electrons from Ge allow the P ions between the two adjacent layers to form strong covalent bonds, promoting the structural phase transition from a layered monoclinic structure to a three-dimensionally coordinated monoclinic lattice as described above. We have shown these two structures schematically in Fig. 3i, j.

### Quasi-2D superconductivity in pressurized thin flakes

**Fig. 4:**
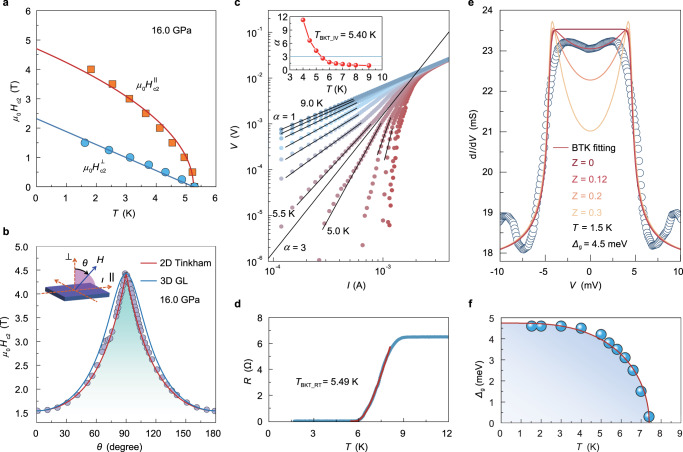
Quasi-2D superconductivity and Andreev reflection in pressurized GeP thin flakes. **a** Temperature dependence of ${H}_{{{{{{\rm{c}}}}}}2}^{\parallel }$ (red squares) and ${H}_{{{{{{\rm{c}}}}}}2}^{\perp }$ (blue circles) at 16.0 GPa in a GeP thin flake (sample 3). Solid lines are the fitted ${H}_{{{{{{\rm{c}}}}}}2}^{\perp }\left(T\right)$ and ${H}_{{{{{{\rm{c}}}}}}2}^{\parallel }\left(T\right)$ curves based on Eq. (1) and Eq. (2), indicating quasi**-**2D superconductivity therein. **b** Angular dependence of ${H}_{{{{{{\rm{c}}}}}}2}\left(\theta \right)$ in sample 3 at 1.5 K and 16.0 GPa. The inset schematically displays the definition of $\theta$, which is the angle between the normal direction of the sample surface and the applied magnetic field. The ${H}_{{{{{{\rm{c}}}}}}2}\left(\theta \right)$ fitting with the 2D Tinkham model ${\left({H}_{{{{{{\rm{c}}}}}}2}(\theta ){{\sin }}\theta /{H}_{{{{{{\rm{c}}}}}}2}^{\parallel }\right)}^{2}+\left|({H}_{{{{{{\rm{c}}}}}}2}\left(\theta \right){{\cos }}\theta )/{H}_{{{{{{\rm{c}}}}}}2}^{\perp }\right |=1$ (red curve) and 3D GL model ${\left({H}_{{{{{{\rm{c}}}}}}2}\left(\theta \right){{\sin }}\theta /{H}_{{{{{{\rm{c}}}}}}2}^{\parallel }\right)}^{2}+{\left({H}_{{{{{{\rm{c}}}}}}2}(\theta ){{\cos }}\theta /{H}_{{{{{{\rm{c}}}}}}2}^{\perp }\right)}^{2}=1$ (blue curve) clearly indi**c**ates that our experimental data can be well described with a 2D superconductivity scenario based on the 2D Tinkham model. The blue-shaded area corresponds to the superconducting state. **c** The BKT fitting based on $V\propto {I}^{\alpha }$ of sample 1 at 17.2 GPa, where the$\,I\left(V\right)$ relations are presented on a log-log scale. The inset displays the power-law exponent $\alpha$ as a function of temperature, and the BKT transition temperature is estimated to be ${T}_{{{{{{\rm{BKT}}}}}}\_{{{{{\rm{IV}}}}}}}$ = 5.40 K as $\alpha$ = 3. **d** The BKT fitting (red line) based on Eq. (3) for $R\left(T\right)$ of sample 1 at 17.2 GPa, where ${T}_{{{{{{\rm{BKT}}}}}}\_{{{{{\rm{RT}}}}}}}$ = 5.49 K. **e** High-pressure Andreev reflection based on a GeP/graphite heterostructure (sample 4) within DAC at 1.5 K. The curves are fitted by the BTK model with different *Z* values of 0, 0.12, 0.2, and 0.3, in which the curve of *Z* = 0.12 is the best fit. The obtained superconducting gap ${\varDelta }_{{{{{{\rm{g}}}}}}}$ is 4.5 meV. **f** The superconducting gap as a function of temperature. The blue balls are data obtained from Supplementary Fig. 18d, and the red curve is the fitting result based on the Bardeen–Cooper–Schrieffer model. The blue-shaded area corresponds to the superconducting state.

Interestingly, the pressure-induced superconductivity in GeP thin flakes can exhibit a quasi-2D nature where the Cooper pairs are spatially confined in a limited thickness of the superconducting region (more discussion in Supplementary Figs. 14 and 15). Experimentally, the in-plane upper critical field ${H}_{{{{{{\rm{c}}}}}}2}^{\parallel }$ can be well described by the phenomenological 2D GL model,

2 $${\mu }_{0}{H}_{{{{{{\rm{c}}}}}}2}^{\parallel }\left(T\right)=\frac{\sqrt{12}{\phi }_{0}}{2\pi {\xi }_{{{{{{\rm{GL}}}}}}}\left(0\right){d}_{{{{{{\rm{SC}}}}}}}}\sqrt{1-\frac{T}{{T}_{{{{{{\rm{c}}}}}}}}}$$

where ${d}_{{{{{{\rm{SC}}}}}}}$ denotes the effective superconducting thickness. Figure 4a shows the ${H}_{{{{{{\rm{c}}}}}}2}^{\perp }$ and ${H}_{{{{{{\rm{c}}}}}}2}^{\parallel }$ as a function of temperature for sample 3 (thin flake with the thickness of ~29 nm) at 16.0 GPa (see more results under higher pressures in Supplementary Fig. 16). Therefore, the quasi-2D nature of superconductivity in GeP can be confirmed based on the excellent fitting of our experimental data to the square-root relationship in Eq. (2). Figure 4b displays the angle-dependent ${H}_{{{{{{\rm{c}}}}}}2}\left(\theta \right)$ at 1.5 K under the same pressure, where $\theta$ is defined as the angle between the normal direction of the sample surface and the applied magnetic field. One can see that the ${H}_{{{{{{\rm{c}}}}}}2}\left(\theta \right)$ curve exhibits a cusp-like peak near $\theta$ = 90° and can be well described with the 2D Tinkham model^22^ (red curve), remarkably distinct from the 3D anisotropic GL model^23^ with a rounded peak (blue curve). Unlike those 2D superconducting behavior observed in interfacial superconductivity^24–26^ and ion-gated surface superconductivity^27–29^, our observations provide the first successful demonstration of realizing quasi**-**2D superconductivity in the metastable phases of superconducting materials under high pressure.

The observed quasi**-**2D superconductivity in our experiment can be well explained by BKT theory^24^. Here, two analysis methods for the BKT transition are applied to our fittings: one is based on ${V}\propto {I}^{\alpha }$ ($\alpha$ is the power-law exponent) and the other is based on the following equation^30–32^,

3 $$R\left(T\right)={R}_{0}\,{{\exp }}\,\left(-\beta {t}^{-\frac{1}{2}}\right),\,t=T/{T}_{{{{{{\rm{BKT}}}}}}}-1$$

where ${R}_{0}$ and $\beta$ are the fitting parameters depending on the material properties. As shown in Fig. 4c, the $I\left(V\right)$ profiles at temperatures near ${T}_{{{{{{\rm{c}}}}}}}$ (more details in Supplementary Fig. 17) obey the power-law relations, and the BKT transition occurs at a temperature ${T}_{{{{{{{\rm{BKT}}}}}}}\_{{{{{\rm{IV}}}}}}}$ where $V\propto {I}^{3}$. The corresponding ${T}_{{{{{{\rm{BKT}}}}}}\_{{{{{\rm{IV}}}}}}}$ is found to be 5.40 K at 17.2 GPa, as plotted in the inset of Fig. 4c. As displayed in Fig. 4d, the BKT transition temperature ${T}_{{{{{{\rm{BKT}}}}}}\_{{{{{\rm{RT}}}}}}}$ can be obtained from the $R\left(T\right)$ curve with Eq. (3). The obtained ${T}_{{{{{{\rm{BKT}}}}}}\_{{{{{\rm{RT}}}}}}}$ $=$ 5.49 K is almost the same as ${T}_{{{{{{\rm{BKT}}}}}}\_{{{{{\rm{IV}}}}}}}\,=$ 5.40 K, which confirms the reliability of our BKT analyses for the experimental data. These observed features of the BKT transition provide further strong evidence for the quasi**-**2D nature of the superconductivity in pressurized GeP thin flakes.

### Superconducting gap features from Andreev reflection

To understand the superconducting gap features of the pressurized GeP flakes, we measured the high-pressure Andreev reflection of differential conductance ${{{{{\rm{d}}}}}}I/{{{{{\rm{d}}}}}}V$ across a planar junction of a GeP/graphite heterostructure (sample 4). One can see a broad peak in differential conductance near zero bias at 1.5 K, as shown in Fig. 4e (for more details, see Supplementary Fig. 18), which can be well-fitted by the Blonder–Tinkham–Klapwijk (BTK) model^33^ with a superconducting gap ${\varDelta }_{{{{{{\rm{g}}}}}}}$ about 4.5 meV and a dimensionless barrier strength *Z* value about 0.12. Such a small *Z* value indicates that our measured tunneling spectra dominate the Andreev reflection regime, as frequently observed in superconductor/metal junction^34–36^ and point-contact spectroscopy^37–43^. The temperature-dependent superconducting gap values (obtained from Supplementary Fig. 18d) can be well fitted by using the empirical relation ${\varDelta }_{{{{{{\rm{g}}}}}}}\left(T\right)={\varDelta }_{{{{{{\rm{g}}}}}}}\left(0\right)\,{{\tanh }}\,\left(1.74\sqrt{({T}_{{{{{{\rm{c}}}}}}}-T)/T}\right)$ as shown in Fig. 4f, which might suggest that the observed superconductivity has a Bardeen-Cooper-Schrieffer-like origin associated with Fermi surface nesting in the band structure. Our results on high-pressure Andreev reflection provide a new direct probe to understand the gap information and the pairing mechanism of such pressurized superconducting states within DAC.

## Discussion

Combining high-pressure transport measurements with first-principles calculations, we demonstrated the existence of a delicate but impactful valence-skipping state in GeP. Such a valence-skipping phenomenon can be described by the Hubbard model with negative-*U* interactions^7–9^, offering opportunities for understanding negative-*U* pairing superconductivity and strongly-correlated charge Kondo effect^5,44^. Meanwhile, the high pressure was shown to be capable of driving this valence-skipping system from a van der Waals compound to a three-dimensional covalent superconductor. Such a covalent nature of the pressurized GeP flake provides us with a brand-new covalent superconductor platform beyond heavily-doped diamond and silicon^45,46^. Our findings offer a new playground to study the quantum criticality and emergent quantum phenomena arising from valence-skipping-like charge fluctuations in spatially confined correlated electronic systems.

## Methods

### Crystal growth and STEM measurements

Single crystals of GeP were synthesized by the temperature gradient method in a cubic high-pressure apparatus (ZN-420). The mixture of germanium powder (Alfa Aesar, 99.999%) and phosphorous powder (Alfa Aesar, 99.999%) with a mole ratio of 1:1 was compressed into a cylinder and inserted in a hexagonal boron nitride capsule with a diameter of 8 mm. The hexagonal boron nitride crucible was encapsulated in MgO and graphite tubes afterward. Heating the starting materials up to 900 °C with maintaining a pressure of 1.0 GPa for 10 min, high-quality GeP was subsequently obtained by cooling the system to room temperature after turning off the electrical power supply.

The samples for cross-sectional transmission electron microscopy (TEM) were cut with a focused ion beam (FIB, Helios Thermo Fisher) from a bulk GeP crystal, and then transferred onto a TEM half grid with the lift-out technique and further processed into a nanosheet by FIB milling. Scanning transmission electron microscopy (STEM) characterization of the GeP crystal was carried out at 300 kV by using an aberration-corrected FEI Themis Z STEM equipped with four detectors energy dispersive X-ray spectroscopy (EDX). Corresponding high-angle annular dark-field (HAADF) images were acquired using a 24-mrad-probe convergence semi-angle, as well as 60-mrad-inner and 200-mrad-out detector angles.

### Sample preparation and thickness estimation

The samples on the diamond culet of DAC were prepared by a standard dry-transfer process. GeP thin flakes were mechanically cleaved from bulk crystals with polydimethylsiloxane (PDMS), then transferred onto the diamond surface. The pre-patterned Ti/Au electrodes (5/15-nm-thick) on the diamond surface were connected with platinum foil electrodes (4 μm in thickness) for electrical wiring. A thin hexagonal boron nitride flake was then transferred on top of the GeP flake for protection. The whole dry-transfer of flakes and electrode wiring process were performed in a N_2_ glovebox to avoid oxidization to ensure good ohmic contact for GeP flakes. The thickness of GeP flakes was identified by atomic force microscopy (AFM) or by the color contrast of the optical image for flakes. Specifically, to avoid any possible sample degradation when exposed in air during the AFM measurement, we only roughly evaluated the sample thickness according to the color contrast from the optical microscopy images for most exfoliated flakes. For samples that require strict thickness confirmation, the AFM measurements were conducted after the samples were transferred onto the diamond culet of DAC.

### DAC high-pressure technique and electronic transport measurements

The standard high-pressure technique was performed in a screw-pressure-type DAC made of nonmagnetic Cu-Be alloy. The culets of the diamond anvils are 300 μm in diameter, which can provide a high pressure of up to 50.0 GPa. A pre-drilled hole (260 μm in diameter) at the center area (pre-indented from 250 to 40 μm in thickness) of a T301 stainless-steel gasket was used as the sample chamber. A mixture of epoxy and cubic boron nitride powder was pressed into the pre-indented area to isolate the electrodes from the metal gasket. Ruby microballs were loaded into the sample chamber to calibrate the precise value of applied pressure with room-temperature R1 ruby photoluminescence.

The electronic transport measurements were performed in an integrated cryogen-free superconducting magnet system (Teslatron^PT^, Oxford Instruments) with the lowest cooling temperature down to 1.5 K and a maximum magnetic field up to 14 T. The standard four-terminal resistance was obtained by using an AC lock-in amplifier (SR830, Stanford Research Systems) with a frequency of 13 Hz. A homemade high-resolution rotator for DAC can provide an angle resolution better than 0.1° for angle-dependent transport measurements, which provides us with more freedom to rotate samples relative to the direction of the magnetic field and with a unique technical platform to study high-pressure induced superconductivity under a “rotatable” magnetic field.

### High-pressure Raman and XRD measurements

Estimations of the sample quality and the high-pressure Raman spectra were carried out at room temperature through Raman spectroscopy using a combined Raman-Atomic Force Microscopy instrument WITec Alpha 300. The individual spectra were acquired by focusing a 5 mW laser with a wavelength of 532 nm (grating: 1800 g/mm) through a ×50 Olympus objective. The raw data were collected as accumulations of 20 s and five times.

The single crystalline sample was ground into fine powders for high-pressure powder XRD measurements. A symmetric DAC with an opening angle of 60° and culets of 500 μm was employed to generate high pressure for the sample. A T301 stainless steel gasket was pre-indented to 20 GPa and drilled into a 250 μm hole to serve as the sample chamber. Silicone oil was utilized as the pressure-transmitting medium. The R1 ruby fluorescence method was used to calibrate the pressure. In-situ high-pressure powder XRD experiments were carried out using a Rigaku Synergy Custom FR-X with *λ* = 0.7107 Å. An area detector with a pixel size of 100 μm × 100 μm was used to collect diffraction patterns. The distance from the sample to the detector was 60.0 mm. The powder XRD pattern under ambient conditions was obtained on SmartLab with Cu-*K*_α_ radiation (*λ* = 1.5406 Å) using a Rigaku X-ray diffractometer. The GSAS program^47^ was used for data integration and structure refinement.

### First-principles calculation

The electronic and volumetric properties of GeP were calculated within density functional theory using the Perdew–Burke–Ernzerhof exchange-correlation functional revised for solids (PBEsol)^48^ and ultrasoft pseudopotentials as implemented in the VASP program^49,50^. We considered a monoclinic structure with point group symmetry *C*_2h_, which is similar to that reported experimentally at ambient pressure. The effect of external hydrostatic pressure was treated by adding a Pulay stress correction to the stress tensor and then fully optimizing the lattice parameters and atomic positions until the magnitude of the force on all ions becomes less than 0.001 eV/Å. The corresponding Brillouin zone was sampled by a 7 × 3 × 7 *k*-mesh at each pressure. The plane-wave cut-off energy was set to 400 eV, and the total energy convergence was fixed at 10^−8^ eV.

## Supplementary information

Supplementary Information

## Data Availability

Source data are provided with this paper. The authors declare that data generated or analyzed during this study are provided as source data or included in the Supplementary Information. Further data are available from the corresponding authors upon request. Source data are provided with this paper.

## Source data

Source Data

## References

[1] Cava RJ (1988). Superconductivity near 30 K without copper: the Ba_0.6_K_0.4_BiO_3_ perovskite. Nature.

[2] Hinks DG, Richards DR, Dabrowski B, Marx DT, Mitchell AW (1988). The oxygen isotope effect in Ba_0.625_K_0.375_BiO_3_. Nature.

[3] Schneemeyer LF (1988). Growth and structural characterization of superconducting Ba_1–*x*_K_*x*_BiO_3_ single crystals. Nature.

[4] Hase I, Yanagisawa T (2008). Valence skip behaviour in BaBiO_3_ and TlS. Phys. C: Supercond..

[5] Matsushita Y, Bluhm H, Geballe TH, Fisher IR (2005). Evidence for charge Kondo effect in superconducting Tl-doped PbTe. Phys. Rev. Lett..

[6] Kriener M (2020). Evolution of electronic states and emergence of superconductivity in the polar semiconductor GeTe by doping valence-skipping indium. Phys. Rev. Lett..

[7] Varma CM (1988). Missing valence states, diamagnetic insulators, and superconductors. Phys. Rev. Lett..

[8] Scalettar RT (1989). Phase diagram of the two-dimensional negative-*U* Hubbard model. Phys. Rev. Lett..

[9] Moreo A, Scalapino DJ (1991). Two-dimensional negative-*U* Hubbard model. Phys. Rev. Lett..

[10] Taraphder A, Coleman P (1991). Heavy-fermion behavior in a negative-*U* Anderson model. Phys. Rev. Lett..

[11] Hase I, Yanagisawa T (2007). Madelung energy of the valence-skipping compound BaBiO_3_. Phys. Rev. B.

[12] Ren Z (2013). Anomalous metallic state above the upper critical field of the conventional three-dimensional superconductor AgSnSe_2_ with strong intrinsic disorder. Phys. Rev. B.

[13] Wakita T (2017). The electronic structure of Ag_1−*x*_Sn_1+*x*_Se_2_ (*x* = 0.0, 0.1, 0.2, 0.25 and 1.0). Phys. Chem. Chem. Phys..

[14] Hertz JA (1976). Quantum critical phenomena. Phys. Rev. B.

[15] Aharony A, Auerbach A (1993). Multicritical phase diagram and random field effects in superconducting bismuthates. Phys. Rev. Lett..

[16] Alexandrov AS, Ranninger J, Robaszkiewicz S (1986). Bipolaronic superconductivity: thermodynamics, magnetic properties, and possibility of existence in real substances. Phys. Rev. B.

[17] Rice TM, Sneddon L (1981). Real-space and $$\vec{k}$$-space electron pairing in BaPb_1−*x*_Bi_*x*_O_3_. Phys. Rev. Lett..

[18] Kittel, C. Introduction to Solid State Physics (John Wiley & Sons, New York, Ed. 8th, 2005).

[19] Zeng T (2021). In-situ templating growth of homeostatic GeP nano-bar corals with fast electron-ion transportation pathways for high-performance Li-ion batteries. Angew. Chem. Int. Ed..

[20] Smiles MJ (2021). Ge 4*s*^2^ lone pairs and band alignments in GeS and GeSe for photovoltaics. J. Mater. Chem. A.

[21] Lee K, Synnestvedt S, Bellard M, Kovnir K (2015). GeP and (Ge_1−*x*_Sn_*x*_)(P_1−*y*_Ge_*y*_) (*x* ≈ 0.12, *y* ≈ 0.05): Synthesis, structure, and properties of two-dimensional layered tetrel phosphides. J. Solid State Chem..

[22] Tinkham M (1963). Effect of fluxoid quantization on transitions of superconducting films. Phys. Rev..

[23] Tinkham, M. Introduction to Superconductivity (Dover, New York, Ed. 2nd, 2004).

[24] Reyren N (2007). Superconducting interfaces between insulating oxides. Science.

[25] Kim M, Kozuka Y, Bell C, Hikita Y, Hwang HY (2012). Intrinsic spin-orbit coupling in superconducting *δ*-doped SrTiO_3_ heterostructures. Phys. Rev. B.

[26] Reyren N (2009). Anisotropy of the superconducting transport properties of the LaAlO_3_/SrTiO_3_ interface. Appl. Phys. Lett..

[27] Saito Y, Nojima T, Iwasa Y (2017). Highly crystalline 2D superconductors. Nat. Rev. Mater..

[28] Saito Y (2015). Superconductivity protected by spin–valley locking in ion-gated MoS_2_. Nat. Phys..

[29] Saito Y, Kasahara Y, Ye J, Iwasa Y, Nojima T (2015). Metallic ground state in an ion-gated two-dimensional superconductor. Science.

[30] Kosterlitz JM, Thouless DJ (1973). Ordering, metastability and phase transitions in two-dimensional systems. J. Phys. C: Solid State Phys..

[31] Kosterlitz JM (1974). The critical properties of the two-dimensional xy model. J. Phys. C: Solid State Phys..

[32] Halperin BI, Nelson DR (1979). Resistive transition in superconducting films. J. Low. Temp. Phys..

[33] Blonder GE, Tinkham M, Klapwijk TM (1982). Transition from metallic to tunneling regimes in superconducting microconstrictions: Excess current, charge imbalance, and supercurrent conversion. Phys. Rev. B.

[34] Lee G-H (2017). Inducing superconducting correlation in quantum Hall edge states. Nat. Phys..

[35] Efetov DK (2015). Specular interband Andreev reflections at van der Waals interfaces between graphene and NbSe_2_. Nat. Phys..

[36] Li J (2020). Superconducting proximity effect in a transparent van der Waals superconductor-metal junction. Phys. Rev. B.

[37] Lee S (2019). Perfect Andreev reflection due to the Klein paradox in a topological superconducting state. Nature.

[38] Daghero D (2012). Strong-coupling *d*-wave superconductivity in PuCoGa_5_ probed by point-contact spectroscopy. Nat. Commun..

[39] Tortello M (2010). Multigap superconductivity and strong electron-boson coupling in Fe-based superconductors: a point-contact Andreev-reflection study of Ba(Fe_1–*x*_Co_*x*_)_2_As_2_ single crystals. Phys. Rev. Lett..

[40] Laube F, Goll G, Löhneysen HV, Fogelström M, Lichtenberg F (2000). Spin-triplet superconductivity in Sr_2_RuO_4_ probed by Andreev reflection. Phys. Rev. Lett..

[41] Sheet G, Mukhopadhyay S, Raychaudhuri P (2004). Role of critical current on the point-contact Andreev reflection spectra between a normal metal and a superconductor. Phys. Rev. B.

[42] Aggarwal L (2016). Unconventional superconductivity at mesoscopic point contacts on the 3D Dirac semimetal Cd_3_As_2_. Nat. Mater..

[43] Wang H (2016). Observation of superconductivity induced by a point contact on 3D Dirac semimetal Cd_3_As_2_ crystals. Nat. Mater..

[44] Dzero M, Schmalian J (2005). Superconductivity in charge Kondo systems. Phys. Rev. Lett..

[45] Ekimov EA (2004). Superconductivity in diamond. Nature.

[46] Blase X, Bustarret E, Chapelier C, Klein T, Marcenat C (2009). Superconducting group-IV semiconductors. Nat. Mater..

[47] Larson, A. C. & Von Dreele, R. B. General Structure Analysis System (GSAS). *Los Alamos National Laboratory Report LAUR*. 86–748 (2000).

[48] Perdew JP (2008). Restoring the density-gradient expansion for exchange in solids and surfaces. Phys. Rev. Lett..

[49] Kresse G, Furthmüller J (1996). Efficient iterative schemes for ab initio total-energy calculations using a plane-wave basis set. Phys. Rev. B.

[50] Kresse G, Joubert D (1999). From ultrasoft pseudopotentials to the projector augmented-wave method. Phys. Rev. B.

