# Supplementary material for: Valence-skipping and quasi-two-dimensionality of superconductivity in a van der Waals insulator
Source: Nat Commun. 2022 Nov 14;13:6938. doi: 10.1038/s41467-022-34726-3 (PMC9663542; doi:10.1038/s41467-022-34726-3)
Supplement: Supplementary file 1 — Supplementary Information [file 41467_2022_34726_MOESM1_ESM.pdf]

Supplementary Information for

Valence-skipping and Quasi-two-dimensionality of Superconductivity in a van der Waals Insulator

Caorong Zhang[1,2†], Junwei Huang[1,3,4†], Kun Zhai[5,6†], Keivan Akhtari[7†], Zhiwei Shen[5,6], Lingyi Ao[1], Zeya Li[1], Feng Qin[1,3,4], Yukai Chang[5,6], Ling Zhou[1], Ming Tang[1,2], Xueting Dai[1], Caiyu Qiu[1,3,4], Yi Zhang[2], Lin Wang[5,6]*, Zhongyuan Liu[5,6], Yongjun Tian[5,6], Mohammad Saeed Bahramy[8,9]*, Hongtao Yuan[1,3,4]*

[1] *National Laboratory of Solid State Microstructures & College of Engineering and Applied Sciences, Nanjing University, Nanjing 210093, China.*

[2] *School of Physics, Nanjing University, Nanjing 210093, China.*

[3] *Collaborative Innovation Center of Advanced Microstructures, Nanjing University, Nanjing 210093, China.*

[4] *Jiangsu Key Laboratory of Artificial Functional Materials, Nanjing University, Nanjing 210093, China.*

[5] *Center for High Pressure Science, Yanshan University, Qinhuangdao 066004, China.*

[6] *State Key Laboratory of Metastable Materials Science & Technology, Yanshan University, Qinhuangdao 066004, China.*

[7] *Department of Physics, University of Kurdistan, Sanandaj 416, Iran.*

[8] *Department of Physics and Astronomy, The University of Manchester, Oxford Road Manchester M13 9PL, United Kingdom.*

[9] *School of Natural Sciences, The University of Manchester, Oxford Road Manchester M13 9PL, United Kingdom.*

[†]These authors contributed equally to this work.

Outline:

1. **Structural characterization of GeP crystals**

2. **Reproducibility for pressure-induced dome-like-shaped superconductivity**

3. **Band gap closing under pressure and the evolution of the Hall coefficient**

4. **Details of the upper critical magnetic field H_{c2} and in-plane coherence length $\xi_{GL}(0)$**

5. **High-pressure XRD, Raman spectra and structure evolution**

6. **Evolution of the Fermi surface of GeP under pressure**

7. **Band structure along the stacking direction and thickness-dependent dimensionality of superconductivity in pressurized GeP**

8. **Experimental confirmation of the quasi-2D nature of SC I and SC II superconducting phases**

9. **$I(V)$ characteristics for BKT analysis under pressure**

10. **High-pressure differential conductance spectra of Andreev reflection**

1. Structural characterization of GeP crystals

To verify the phase of the sample, we performed powder X-ray diffraction (XRD) measurements of ground GeP crystals under ambient conditions. As shown in the Rietveld analysis of the powder XRD pattern in Supplementary Fig. 1, the blue open circles (labelled as "Experimental XRD data") represent the obtained experimental XRD data, and the red solid line (labelled as "Calculated XRD pattern") represents the Rietveld refinement results for analysing the crystal lattice with General Structure Analysis System (GSAS) software[1]. The black vertical bars (labelled as "Theoretical peak position") represent the theoretical peak positions of monoclinic GeP by the GSAS software. By comparing the experimental XRD data with the simulated diffraction peaks of monoclinic GeP, we can label all the experimental XRD peaks with (hkl) indexes. One can see that all Bragg diffraction peaks in the XRD spectrum can be well indexed by a monoclinic crystal structure ($C2/m$ space group) with lattice parameters a = 14.906 Å, b = 3.653 Å, c = 9.180 Å, $\alpha = \gamma$ = 90°, and β = 100.123°, which is consistent with a previous report[2]. Note that the good weighted profile R-factor R_{wp} = 6.6% obtained from the Rietveld refinement is much better than the well-accepted standard of 10%, and there is no peak for any other type of crystal structure in our spectrum, which directly confirms the purity of the monoclinic GeP phase.

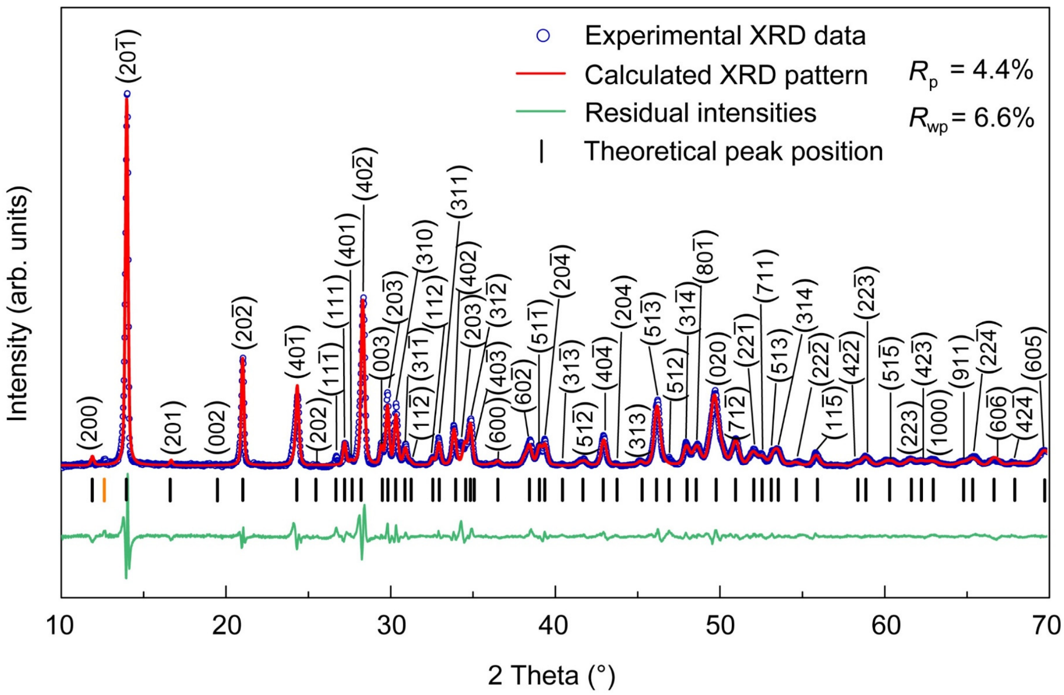

Supplementary Fig. 1 | Powder XRD pattern of ground GeP crystals under ambient conditions. The open circles (Experimental XRD data) represent the obtained experimental XRD data, and the red solid line (Calculated XRD pattern) represents the Rietveld refinement results for analysing the crystal lattice. The green solid line (Residual intensities) at the bottom denotes the residual intensities (the difference between the experimental pattern and Rietveld refined pattern). The black vertical bars (Theoretical peak position) indicate the theoretical peak positions of monoclinic GeP. The orange vertical bar indicates the unidentified peak (may originate from experimental errors). The parameters obtained from the Rietveld refinement results are R_p = 4.4% and R_{wp} = 6.6% (weighted profile). The wavelength of the X-ray is λ = 1.5406 Å.

The STEM was performed to confirm the crystal structure of GeP flakes. Supplementary Fig. 2a–d shows a high-angle annular dark-field (HAADF) image and corresponding EDX elemental maps of a cross-sectional GeP single crystal, indicating a uniform distribution of Ge and P elements in the sample. The selected area electron diffraction pattern (Supplementary

Fig. 2e) of the sample shows one set of diffraction spots, which is well satisfied with that of monoclinic GeP single crystals taken along the [010] zone axis. Supplementary Fig. 2f shows the corresponding atomic-scale HAADF image of the GeP crystal, in which the Ge and P atoms are clearly illustrated owing to the atomic number Z-dependent intensity of the HAADF image. A layer spacing of 6.6 Å matches well with the value of 6.7 Å between the $(20\bar{1})$ planes in the reported GeP monoclinic structure[3].

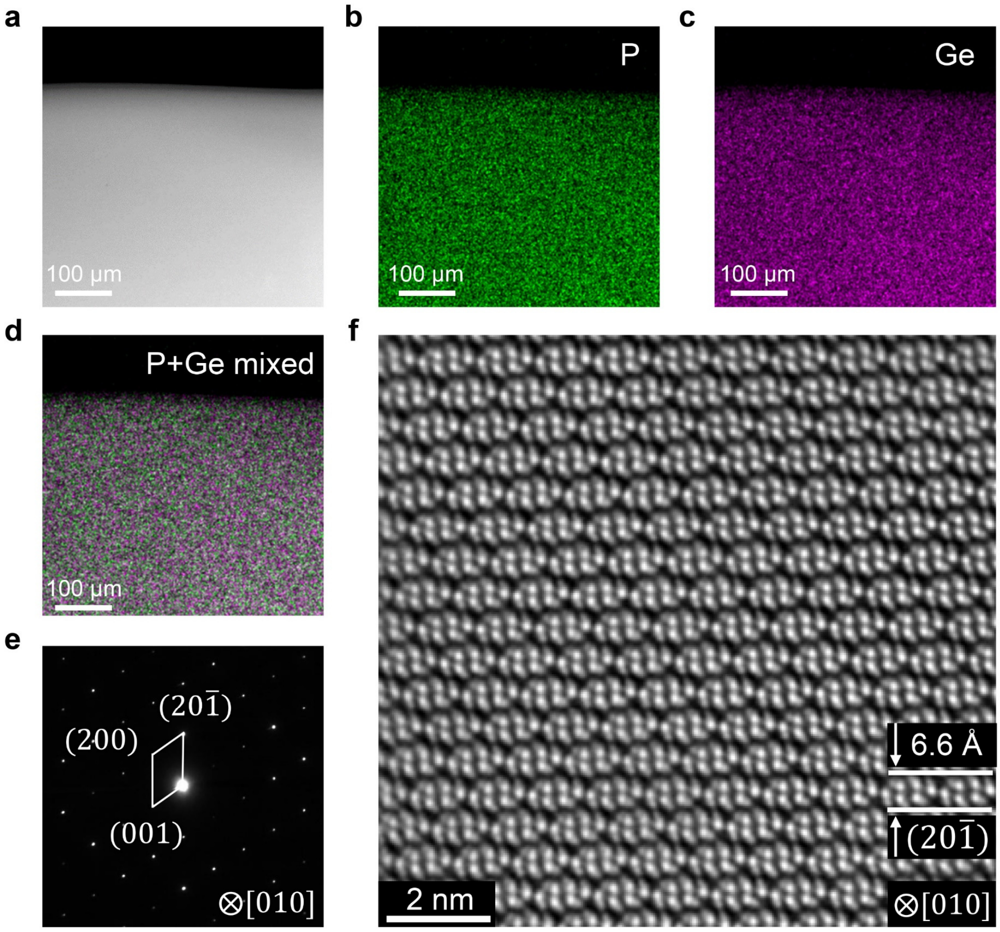

Supplementary Fig. 2 | STEM characterization of GeP crystals. a–d, HAADF image and corresponding EDX mapping of a cross-sectional GeP single crystal. **e**, Selected area electron diffraction pattern of the GeP crystal taken along the [010] zone axis, in which the reciprocal indexes of $(20\bar{1})$, (200) and (001) are labelled. **f**, Atomic-scale HAADF image of the GeP crystal viewed along the [010] direction, and the layer spacing between the $(20\bar{1})$ planes can be confirmed to be 6.6 Å.

2. Reproducibility for pressure-induced dome-like-shaped superconductivity

Similar superconducting phase diagrams on several GeP flakes with different thicknesses (sample 1 > sample 3 > sample 5) can be observed in Supplementary Fig. 3. One can see in Supplementary Fig. 3a (sample 3) that the superconducting state with a resistance drop appears at 12.6 GPa, and the critical temperature T_{c} reaches a maximum value of 8.3 K at 25.0 GPa. The superconducting transition of GeP (sample 5) is shown in Supplementary Fig. 3b. The $T_{\mathrm{c}}(P)$ data also display a similar shape, where T_{c} reaches a maximum value of 7.0 K at 29.2 GPa. The comparison of the phase diagrams of pressurized GeP with different thicknesses is provided in Supplementary Fig. 3c. One can see that, even though the thickness of GeP is various, the superconducting phase diagram shows a similar shape. The T_{c} values of those samples develop with sample thickness, which can serve as a future research project.

The thickness of the measured samples was estimated according to the color contrast of optical images and confirmed by atomic force microscopy (AFM) measurements on a diamond culet. Specifically, to avoid any possible sample degradation when exposed to air during the atomic force microscopy measurement, we only roughly evaluated the sample thickness according to the color contrast from the optical microscopy images of the exfoliated flakes. We found that the sample thickness follows the sequence: sample 1 (hundreds of nanometers) > sample 3 (tens of nanometers) > sample 5 (several nanometers). Importantly, based on experimental requirements, we strictly confirmed the sample thickness with AFM after the sample was transferred onto the diamond culet of DAC, in which sample 3 with quasi-two-dimensional (quasi-2D) superconductivity was strictly confirmed to be 29 nm in thickness.

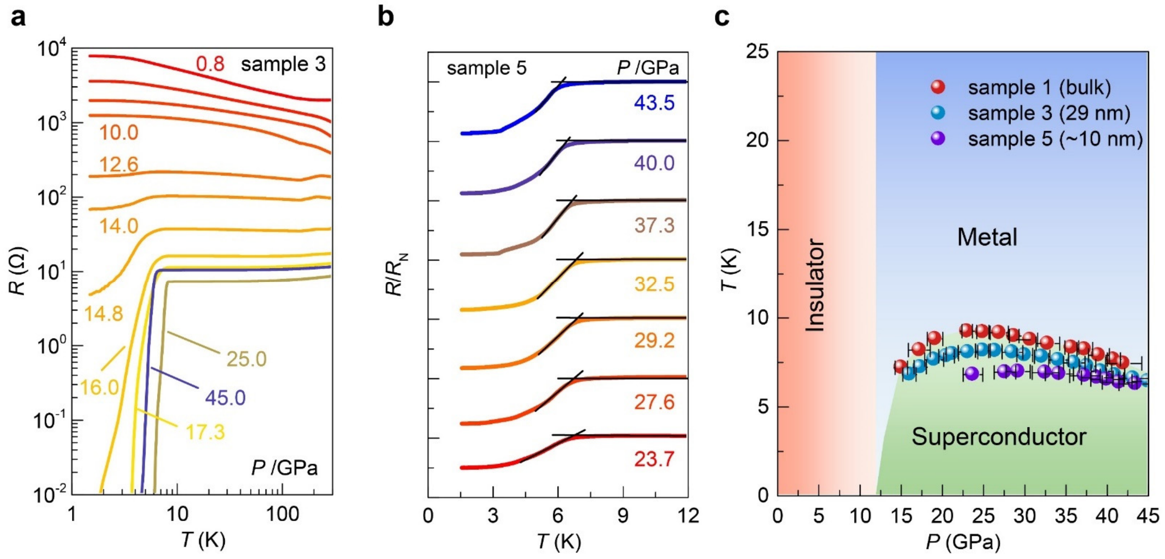

Supplementary Fig. 3 | Pressure-induced superconductivity of GeP flakes with different thicknesses. a, Temperature-dependent resistance of the GeP flake (sample 3) under various pressures from 0.8 to 45.0 GPa. **b**, Normalized resistance R/R_{N} (R_{N}: normal state resistance at 12 K) of ultrathin GeP (sample 5) with applied pressures from 23.7 to 43.5 GPa. **c**, Phase diagram of pressurized GeP with different thicknesses (sample 1 in red, sample 3 in cyan and sample 5 in purple). The insulating phase is shaded in red, the metal phase is shaded in blue, and the superconducting phase is shaded in green. The error bars on pressure are determined by the differences in fluorescence lines at different positions on the same ruby.

3. Band gap closing under pressure and the evolution of the Hall coefficient

To understand the evolution of the band gap of GeP under pressure, we analysed the thermal activation gap based on the $R(T)$ data (Fig. 1f in the main text) via $G(T) \propto e^{-\Delta/(2k_B T)}$ below 15.0 GPa. As shown in the Arrhenius plot of the conductance versus the reciprocal of temperature for each pressure (Supplementary Fig. 4a), one can estimate the activation gap values (Supplementary Fig. 4b) through good linear fittings in the thermally activated region (black dashed lines). Note that the thermal activation gap gradually closes with increasing pressure and fully closes at ~15.0 GPa. This critical pressure is consistent with the calculation results in Fig. 3d of the main text. These observations of the existing activation gap can help us confirm the typical insulating states of GeP at low pressures.

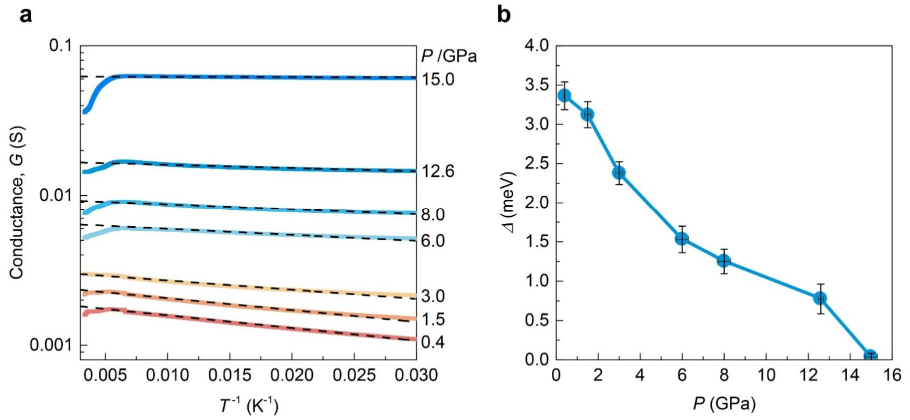

Supplementary Fig. 4 | The thermal activation gaps as a function of pressure. a, Arrhenius plot of the conductance as a function of inverse temperature at various pressures ranging from 0.4 to 15.0 GPa. The dashed black lines are fits of the data based on $G(T) \propto e^{-\Delta/(2k_B T)}$, where Δ is the activation gap and k_B is the Boltzmann constant. **b**, The fitted thermal activation gaps as a function of pressure. Note that the thermal activation gap gradually closes with increasing pressure and fully closes at ~15.0 GPa. The error bars on the gaps represent the uncertainty arising from determining the linear regime for the fit.

The Hall effect measurements on sample 2 were performed at 1.5 K to understand the evolution of the charge carrier density of GeP with pressure. Actually, the carrier density is deduced from the Hall effect measured at 1.5 K by applying magnetic fields to ±8 T (larger than the H_{c2}). The details of the deduction process are provided as follows.

i. Obtain the raw $R_{xy}(H)$ data

The raw $R_{xy}(H)$ was acquired by sweeping the magnetic field H from +8 T to –8 T and then back to +8 T (larger than the H_{c2}). Then, we obtained the raw $R_{xy}(H)$ data.

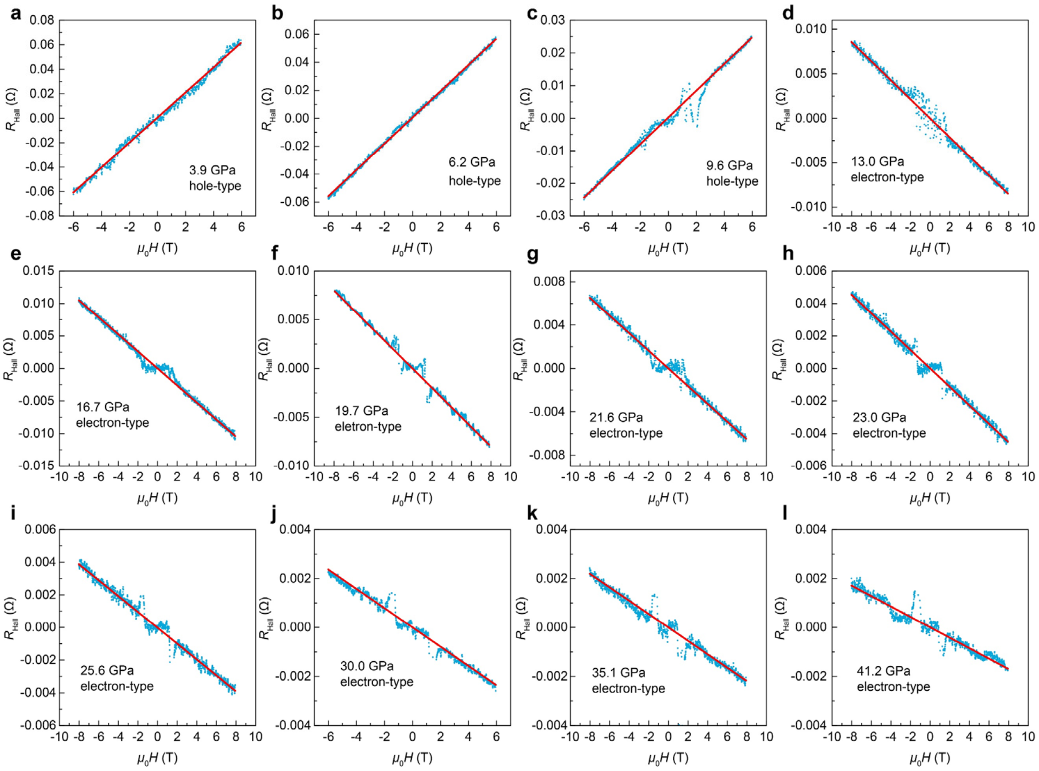

Supplementary Fig. 5 | Hall resistance R_{Hall} obtained by anti-symmetrization and fitted with red linear curves. a–l, The $R_{\mathrm{Hall}}(H)$ data (blue dots) at 1.5 K measured at each pressure of 3.9, 6.2, 9.6, 13.0, 16.7, 19.7, 21.6, 23.0, 25.6, 30.0, 35.1 and 41.2 GPa. The $R_{\mathrm{Hall}}(H)$ data at each pressure can be well fitted with a linear relation (red curves) whose slope corresponds to the major carriers in hole-type (**a**–**c**) and electron-type (**d**–**l**). The Hall coefficient R_{H} is derived by the slope of the fitted line (red line). The carrier density of the GeP sample can be determined by the corresponding Hall coefficient based on the relation $n = \frac{1}{eR_{\mathrm{H}}}$.

ii. Remove the $R_{xx}(H)$ component

Note that the raw $R_{xy}(H)$ data contain the contributions from magnetoresistance $R_{xx}(H)$ and normal Hall resistance $R_{\mathrm{Hall}}(H)$, where $R_{xx}(H)$ $[R_{\mathrm{Hall}}(H)]$ is symmetric (anti-symmetric) with respect to the polarity of the magnetic field. In other words, $R_{xx}(+H) = R_{xx}(-H)$, and $R_{\mathrm{Hall}}(+H) = -R_{\mathrm{Hall}}(-H)$. Therefore, by applying a standard anti-symmetrization procedure, we can remove the symmetric $R_{xx}(H)$ component from the $R_{xy}(H)$ data, and then obtain the $R_{\mathrm{Hall}}(H)$ data (the blue curves in Supplementary Fig. 5). Here, $R_{\mathrm{Hall}}(H) = [R_{xy}(+H) - R_{xy}(-H)]/2$.

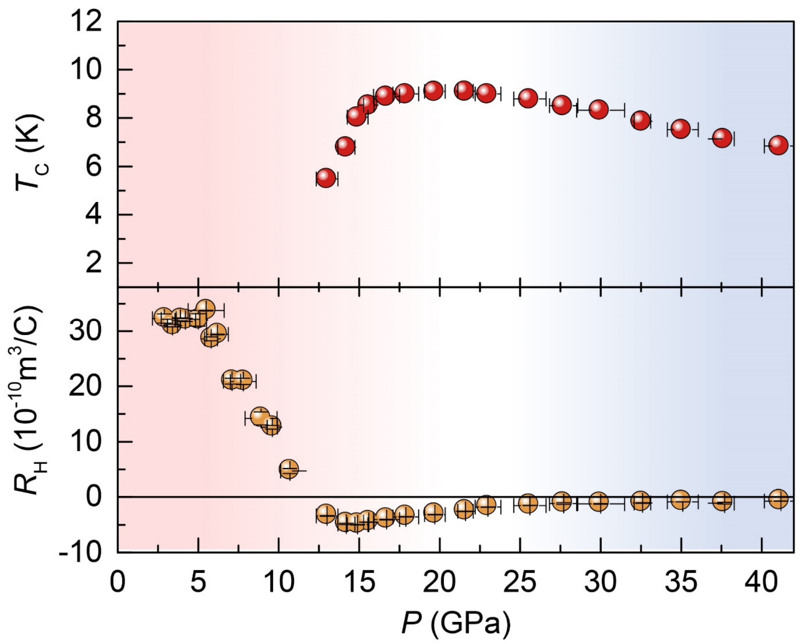

Supplementary Fig. 6 | The evolution of T_c and the Hall coefficient with pressure. The upper panel shows the $T_c(P)$ superconducting phase diagram, and the bottom panel shows the Hall coefficient as a function of pressure. Error bars on the Hall coefficient represent the uncertainty arising from determining the linear regime for the fit. Error bars on pressure are determined by the differences in fluorescence lines at different positions on the same ruby. The red shaded area corresponds to the quasi-2D monoclinic structure phase, and the blue shaded area corresponds to the bulk monoclinic structure phase.

iii. Obtain the R_{H} value

We fit the $R_{\mathrm{Hall}}(H)$ data at magnetic fields above H_{c2} (here, in the range of 3 ~ 8 T) by using a linear relation (the red curves in Supplementary Fig. 5), and we obtain the slope of the linear line, considering the sample thickness, then we obtain the Hall coefficient R_{H}.

iv. Deduce the carrier density

Finally, the carrier density n is deduced from the Hall coefficient R_{H} based on the relation $n = \frac{1}{eR_{\mathrm{H}}}$, where e is the electron charge.

The Hall coefficient as a function of pressure is presented in Supplementary Fig. 6. One can see that the Hall coefficient has a clear sign change from positive to negative as the superconductivity occurs around 13.0 GPa, suggesting that the major carrier type transitions from hole to electron.

Below 13.0 GPa, the Hall coefficient is positive, and for example, the hole carrier density is ~ 2×10^{21} cm^{-3} at 5.0 GPa (Fig. 2b). Such hole-type major carriers for the Hall effect are contributed from the valence band of the P-3*p* orbital at the Γ point. Above 13.0 GPa, the Hall coefficient is negative, suggesting that the major carriers are electrons. Such electron-type major carriers for electrical transport are contributed from the conduction band of the Ge-4*p* orbital at the N point.

4. Details of the upper critical magnetic field H_{c2} and in-plane coherence length

The resistance as a function of temperature and magnetic field was plotted to analyse the upper critical field and in-plane coherence length. Taking the results of 17.2 GPa (sample 1) as an example, Supplementary Fig. 7a, b shows the $R(T)$ curves under various magnetic fields and the $R(H)$ curves at various temperatures, where the magnetic fields are applied perpendicular to the sample surface. Supplementary Fig. 7c displays the $H_{c2}(T)$ curve of the superconducting GeP flake at 17.2 GPa. The T_c and H_{c2} values obtained from two different upper critical field measurements are consistent. One can obtain the H_{c2} value at zero temperature and the in-plane Ginzburg-Landau (GL) coherence length $\xi_{GL}(0)$ by fitting the data to equation (1) in the main text.

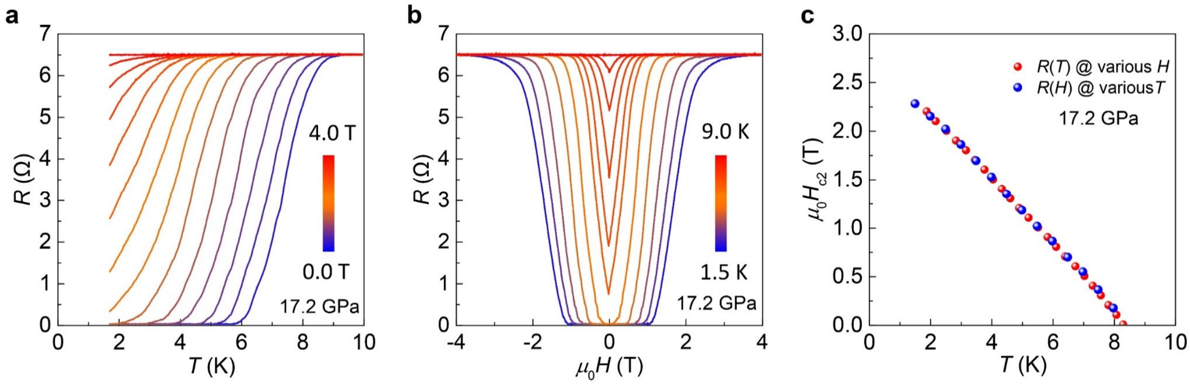

Supplementary Fig. 7 | Upper critical field measurements for superconducting GeP at 17.2 GPa. a, $R(T)$ curves of GeP flake under various magnetic fields ranging from 0 to 4.0 T. **b**, $R(H)$ curves of GeP flake at various temperatures ranging from 1.5 to 9.0 K. **c**, $H_{c2}(T)$ of the superconductivity of the GeP flake at 17.2 GPa, where red and blue balls are obtained from the T_c and H_{c2} values from the $R(T)$ curves of the GeP flake under various magnetic fields in (**a**) and the $R(H)$ curves of the GeP flake under various temperatures in (**b**).

Supplementary Fig. 8a, b shows the evolution of the $R(H)$ curves with pressure at 2.0 K and the evolution of the $H_{c2}(T)$ values with pressure (sample 1). The pressure-dependent $H_{c2}(T = 0.0\ \mathrm{K})$ values are obtained based on the analysis with equation (1) in the main text, as shown in Supplementary Fig. 8c. One can see a sudden decrease in $H_{c2}(T = 0.0\ \mathrm{K})$ values around P_{M}, similar to the $H_{c2}(T = 2.0\ \mathrm{K})$ evolution with pressure, indicating the structural phase transition near P_{M}. Note that the $H_{c2}(T = 0.0\ \mathrm{K})$ values are much smaller than the Pauli paramagnetic limit $\mu_0 H_{\mathrm{P}} = 1.84 T_{\mathrm{c}}$ for the Bardeen-Cooper-Schrieffer superconductor in the whole pressure range, which can support the analysis mentioned above since $H_{c2}(T)$ is dominated by the orbital limit and is irrelevant to Pauli pair breaking.

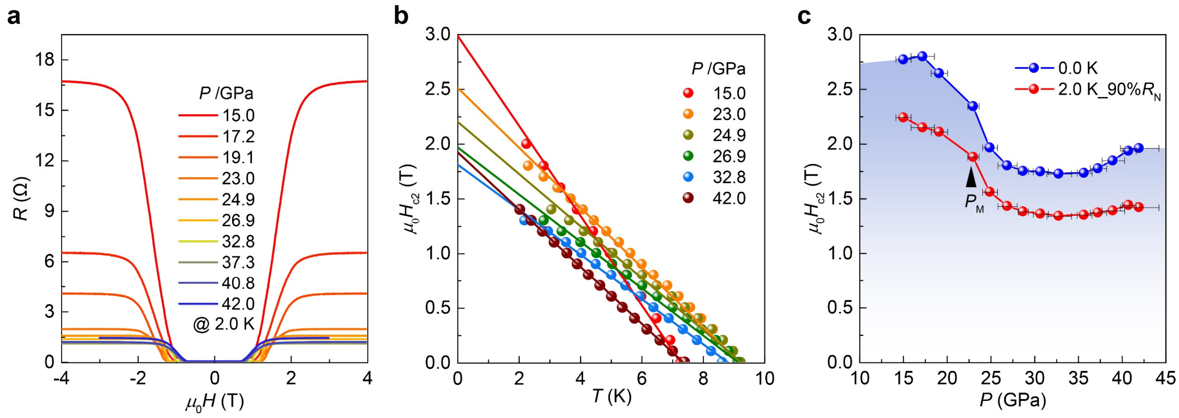

Supplementary Fig. 8 | Pressure-dependent upper critical magnetic field H_{c2} at 2 K. a, Magnetoresistance measured at 2.0 K under various pressures of 15.0, 17.2, 19.1, 23.0, 24.9, 26.9, 32.8, 37.3, 40.8 and 42.0 GPa. **b**, Temperature-dependent H_{c2} under various pressures of 15.0, 23.0, 24.9, 26.9, 32.8 and 42.0 GPa. **c**, Pressure-dependent upper critical field, in which the red balls of H_{c2} values are obtained from the magnetoresistance measured at 2.0 K and defined as 90% of the normal state resistance (R_{N}), while the blue balls of H_{c2} values are obtained from the fitting to equation (1) in the main text. The black arrow marks the pressure P_{M} of the structure phase transition. The blue shaded area corresponds to the superconducting state. The error bars on pressure are determined by the differences in fluorescence lines at different positions on the same ruby.

5. High-pressure XRD, Raman spectra and structure evolution

The simulation of the XRD spectrum of GeP powder is performed by the GSAS software to refine the crystal parameters and label the observed Bragg peaks. GeP has a monoclinic crystal structure with the $C2/m$ space group, which provides the theoretical main Bragg peaks shown in Supplementary Fig. 9. One can see that within the observed 2 Theta range from 8 to 32°, there are 52 Bragg peaks with relatively large intensity. The theoretical 2 Theta values of those Bragg peaks match well to the experimental data, as shown in Supplementary Fig. 9.

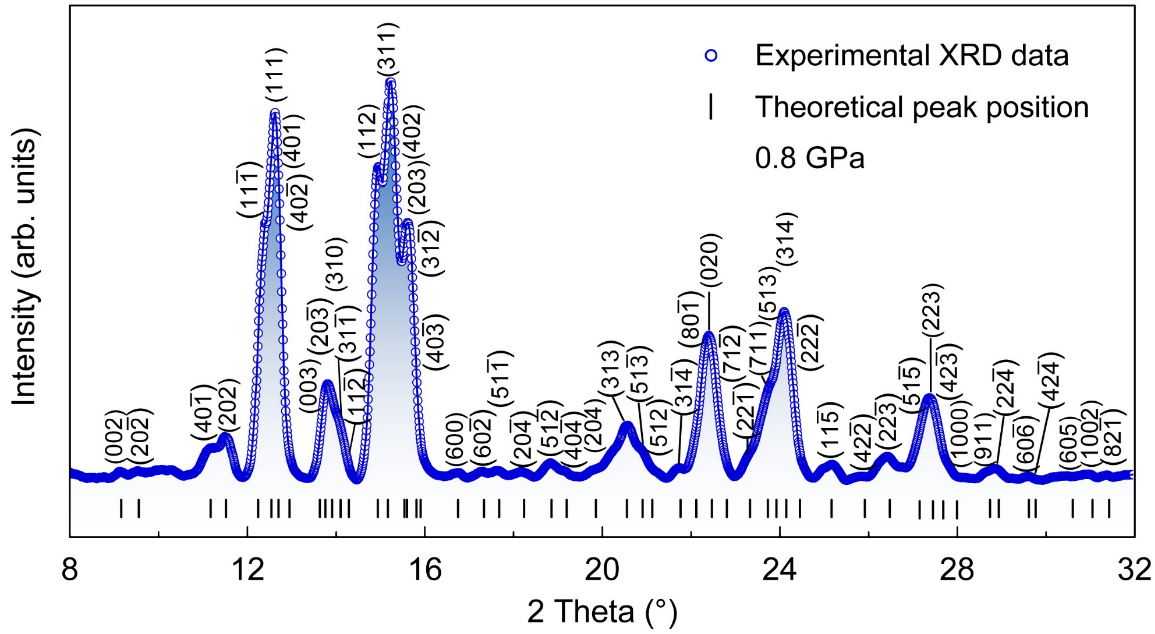

Supplementary Fig. 9 | Peak indexing for the experimental XRD spectrum at 0.8 GPa. The experimental XRD spectrum (open circles) at 0.8 GPa are all labelled by (hkl) indexes according to the theoretical peak positions (black vertical bars) of monoclinic GeP. The wavelength of the X-ray is $\lambda = 0.7107$ Å.

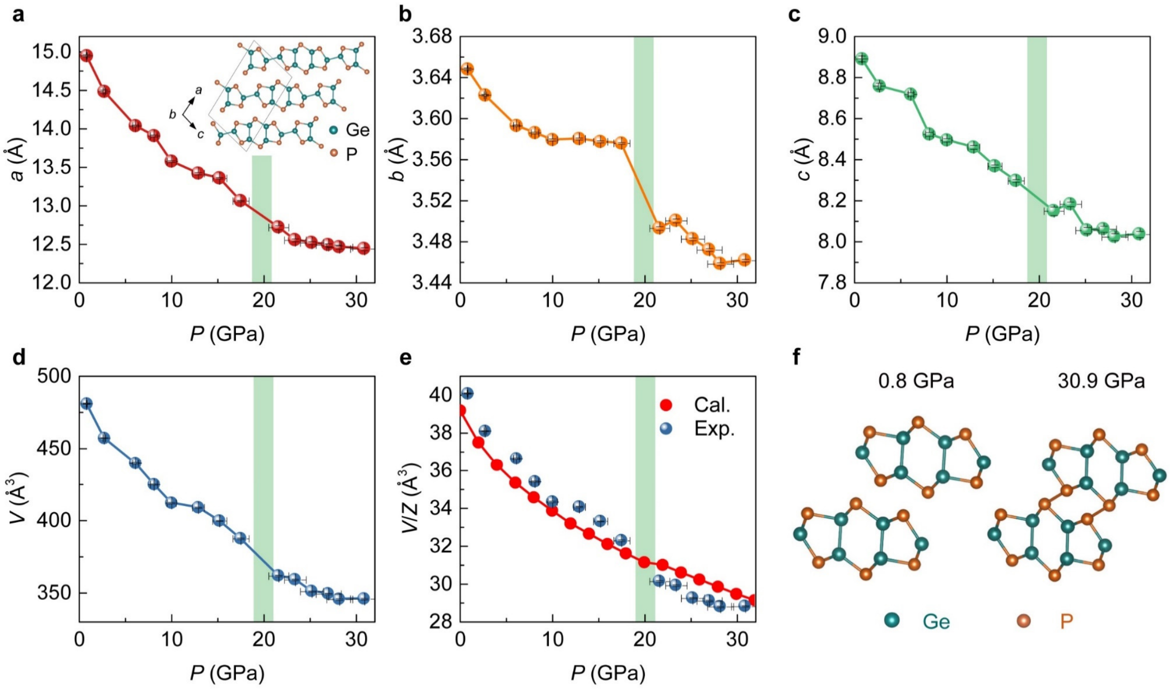

Supplementary Fig. 10 | The evolution of the lattice parameters and the unit-cell volume with pressure obtained from Rietveld refinement results. a–d, The pressure-dependent lattice parameters of *a*, *b* and *c*, and the unit-cell volume *V*. The green area highlights the pressure range in which the 2D to 3D structural phase transition takes place. The inset in panel a shows the schematic crystal structure of GeP (cyan balls for Ge and orange balls for P), in which the black box represents the unit cell of monoclinic GeP and the black arrows represent the corresponding lattice vectors of *a*, *b* and *c*. **e**, Comparison of the experimental (Exp.) and calculated (Cal.) unit-cell volume per formula as a function of pressure. The error bars in **a**–**e** represent the standard deviation. **f**, Schematic illustration of the pressure-induced structural phase transition in GeP crystal structure (cyan balls for Ge and orange balls for P) from a quasi-2D monoclinic structure (0.8 GPa) to a bulk monoclinic structure (30.9 GPa).

To understand the evolution of lattice parameters with respect to pressure, we refined the experimental XRD patterns by using the standard Rietveld method via the GSAS program[1]. For all the measured XRD results under each applied pressure, the experimental XRD peaks

can be indexed by a monoclinic crystal structure with a good weighted profile R-factor R_{wp}, and thus the lattice parameters can be obtained from the Rietveld refinement results, and the evolution of lattice parameters with respect to pressure is shown in Supplementary Fig. 10. One can see in Supplementary Fig. 10a–e that the evolution of the refined lattice parameters with pressure shows a clear jump around P_{M}, which is a signature for the crystal structural phase transition. This critical pressure for the structural phase transition is consistent with the results of the experimental Raman spectra (Fig. 2d in the main text) and first-principles calculations (Fig. 3 in the main text).

Considering the 2D to 3D structural phase transition, the formation of new bonds between the P and P atoms (from two layers of GeP) at high pressure could be the most possible mechanism for this phenomenon. Specifically, we note that the lattice parameter c decreases rather rapidly at pressures lower than 19.2 GPa, while it starts to decrease more slowly at pressures higher than 21.6 GPa, suggesting that a unique structural phase transition occurs at this particular pressure. In such a structural phase transition, our theoretical calculations reveal that the interlayer interaction of adjacent layers of P atoms becomes more pronounced and finally promotes the formation of covalent bonds between the P and P atoms (from two layers of GeP) at high pressure. As a result, two layers of GeP are covalently bonded, causing the structural phase transition from the 2D layered structure to the 3D bulk case in the whole system (two structures schematically shown in Supplementary Fig. 10f).

High-pressure Raman spectral measurements were performed to investigate the lattice vibration modes. It is well known that pressure can effectively modulate the crystal structure, which can be clarified through lattice vibration modes. Based on the theoretical analysis of group theory, the monoclinic GeP lattice structure contains 33 irreducible representations, which can be expressed as $\Gamma = 12\ A_{\mathrm{g}} + 5\ A_{\mathrm{u}} + 6\ B_{\mathrm{g}} + 10\ B_{\mathrm{u}}$, where A_{g} and B_{g} are Raman active modes while A_{u} and B_{u} are infrared active modes.

The Raman spectrum under ambient conditions (Fig. 2d, left panel) exhibits sharp peaks matching those reported spectra well[3,4], indicating that our GeP crystal is of high quality. Supplementary Fig. 11a shows the pressure-dependent Raman spectra obtained at room temperature. Supplementary Fig. 11b shows the enlarged Raman spectra at low pressure. One can see that the B_g^1 peak around 79 cm^{-1} shifts towards larger wavenumbers with increasing pressure, and its intensity is enhanced correspondingly, while the B_g^2 peak around 96 cm^{-1} exhibits redshift behavior. Therefore, the B_g^1 and B_g^2 peaks show a crossover at a critical pressure of ~1.5 GPa. Supplementary Fig. 11c shows the frequency evolution of Raman peaks under pressure. Two important points should be addressed here. First, the frequencies of all vibration modes increase continuously with increased pressure. Second, two new Raman peaks appear above 23.0 GPa (near P_M), verifying the occurrence of the structural phase transition from the layered monoclinic to the bulk monoclinic phase, as described in the main text. The intensity of all Raman peaks dramatically decreases to the noise level at pressures above 30.0 GPa, due to the high electron density of states (DOS) and too strong reflectance from the sample surface to optically detect the Raman signal.

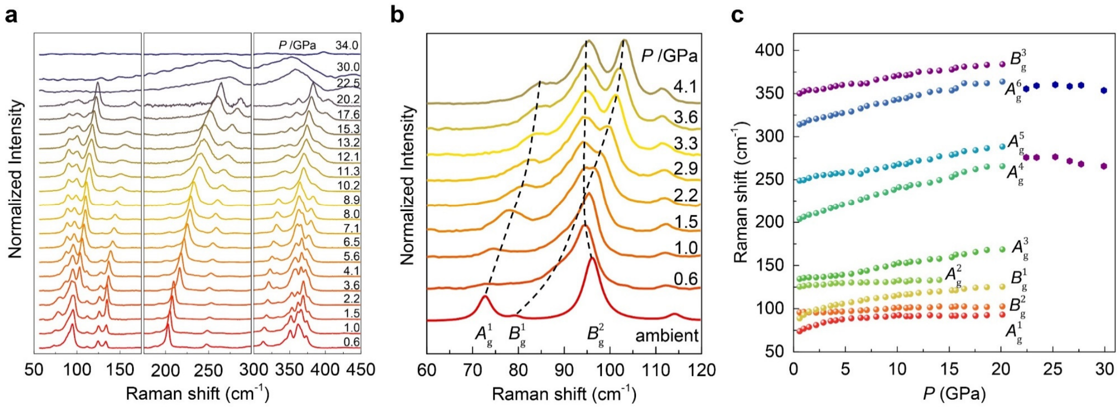

Supplementary Fig. 11 | Raman spectra of GeP under pressure at room temperature. a, Typical evolution of Raman spectra with pressures ranging from 0.6 to 34.0 GPa. **b**, Enlarged Raman spectra at low pressure (below 4.1 GPa). **c**, The evolution of Raman vibration frequencies with pressure of each A_g and B_g Raman active mode.

To exclude the amorphization of the crystal and understand the Raman peak broadening at high pressure, we provided the Raman spectra to confirm the reversibility during the compression and decompression process in the Supplementary Fig. 12a. Specifically, as the pressure increases, two A_g^4 (203 cm^{-1}) and A_g^6 (312 cm^{-1}) peaks evolve into two broad peaks centered at 275 cm^{-1} and 362 cm^{-1} at pressures above ~23.0 GPa, and the intensities of those two broad peaks decrease quickly to the background noise level with further pressure increases. During the decompression process, two broad peaks appear back from the background noise level when the pressure is decreased to 20.2 GPa. After decompressing the pressure back to zero, all the peaks of the monoclinic GeP structure return. Such reversibility of the Raman spectra before compression and after decompression clearly indicates the mechanism of pressure-driven crystal structural phase transition for the appearance of such two broad peaks and rules out the possibility of the amorphization of the crystal.

Correspondingly, the experimental XRD diffraction peaks under pressure (shown in Supplementary Fig. 12b) do not become broad at high pressure, excluding the possibility of crystal structure amorphization under high pressure. Importantly, the Bragg peaks of (111), (313), and (314) are strongly suppressed as the pressure increases to around 23.0 GPa, while the Bragg peaks of (020) and (223) remain when the pressure is higher than 23.0 GPa, directly confirming that superconducting GeP has a crystalline structure under high pressure rather than an amorphous one. Such a crystal structural phase transition at the pressure around 23.0 GPa can be further identified as the formation of covalent bonds between P atoms between adjacent layers, turning the layered monoclinic structure into a three-dimensionally coordinated monoclinic lattice with charge redistribution due to the valence skipping mechanism.

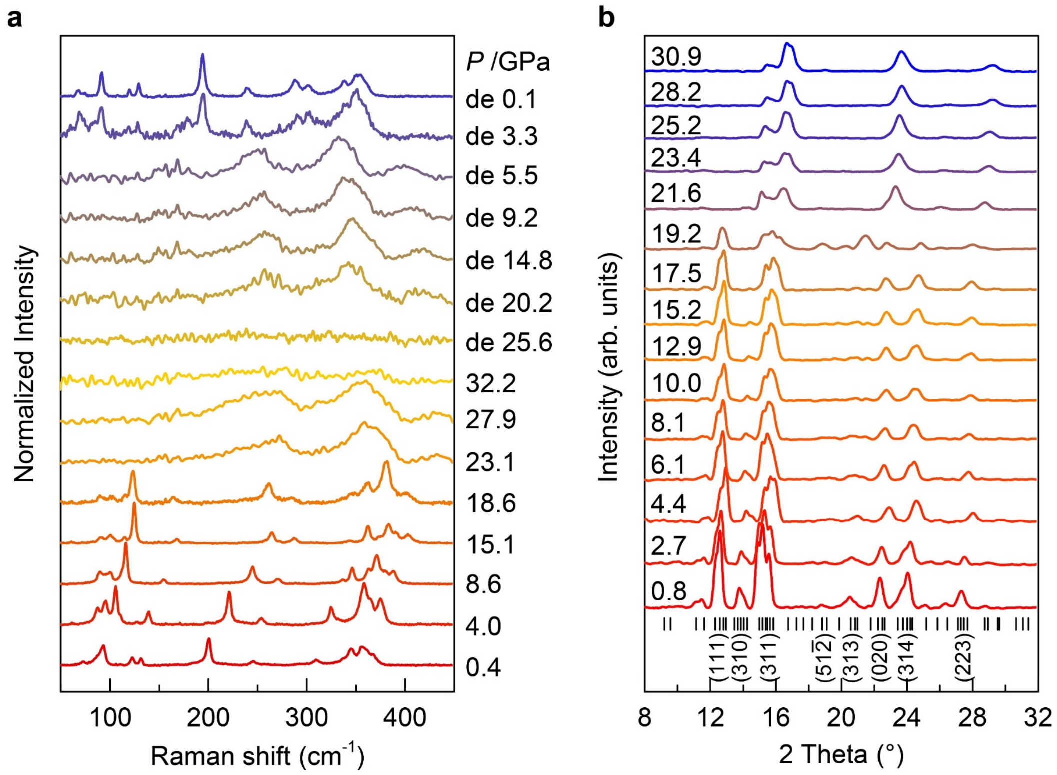

Supplementary Fig. 12 | Raman spectra and XRD patterns of GeP under pressure at room temperature. **a**, Normalized Raman spectra (normalized to the highest peak value) of GeP during the compression at each pressure ranging from 0.4 to 32.2 GPa and decompression process at each pressure ranging from 32.2 to 0.1 GPa. **b**, High-pressure powder XRD patterns under each pressure ranging from 0.8 to 30.9 GPa collected by a Mo-K_α target with $\lambda = 0.7107$ Å, the main (hkl) indexes are labelled according to the theoretical peak positions (black vertical bars) of monoclinic GeP.

6. Evolution of the Fermi surface of GeP under pressure

To understand the evolution of the Fermi surface of GeP, we performed first-principles calculations under different pressures. As indicated in Fig. 3d of the main text, our calculations suggest that GeP below 15.0 GPa is an insulator, implying that it has no energy pocket at the Fermi level. Above 15.0 GPa, however, we do see a series of electron and hole pockets that rapidly grow in size by increasing the pressure. To clarify this, below, we have shown the calculated Fermi surfaces at 18.0 GPa, 28.0 GPa and 38.0 GPa, as shown in Supplementary Fig. 13. As can be seen, just above the insulator-metal phase transition, the system gains small Fermi pockets. The hole pockets, shown in blue, are more localized around the Zone centre, whereas the electron ones, shown in red, are scattered near the Zone boundaries. Nevertheless, they all commonly stretched along the stacking direction due to the quasi-two-dimensionality of GeP. At 28.0 GPa, the hole pockets fully merge and form two cylinders, again parallel to the stacking direction. At 38.0 GPa, these energy pockets have grown so drastically that almost the whole Brillouin zone is filled, signifying the role of pressure in deriving the structural and electronic phase transitions in GeP.

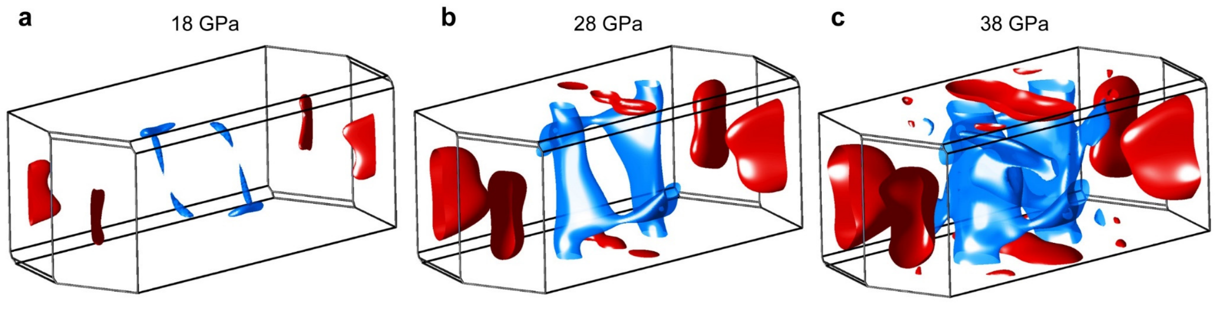

Supplementary Fig. 13 | Evolution of the Fermi surface under pressure. a–c, The calculated Fermi surface of GeP at **a**, 18.0 GPa **b**, 28.0 GPa and **c**, 38.0 GPa. The Fermi surface of the hole and electron pockets are shown in blue and red, respectively.

7. Band structure along the stacking direction and thickness-dependent dimensionality of superconductivity in pressurized GeP

The GeP system shows weak electronic dispersion along the stacking direction, i.e., the Γ–Z direction in the Brillouin zone. As shown in Supplementary Fig. 14, we have compared the band structure of GeP along the Z–Γ–Z direction at 0 and 38.0 GPa. As can be seen, the valence (hole) band remains effectively dispersionless across this pressure range. The conduction (electron) band tends to disperse more steeply at high pressure. Nevertheless, even at 38.0 GPa, when we closely inspect the whole Brillouin zone, we can see that the resulting hole and electron pockets are significantly elongated along the stacking (Γ–Z) direction (see Fig.3f in the main text). This result accordingly confirms the quasi-two-dimensionality of the electronic transport and, thus, superconductivity in this material.

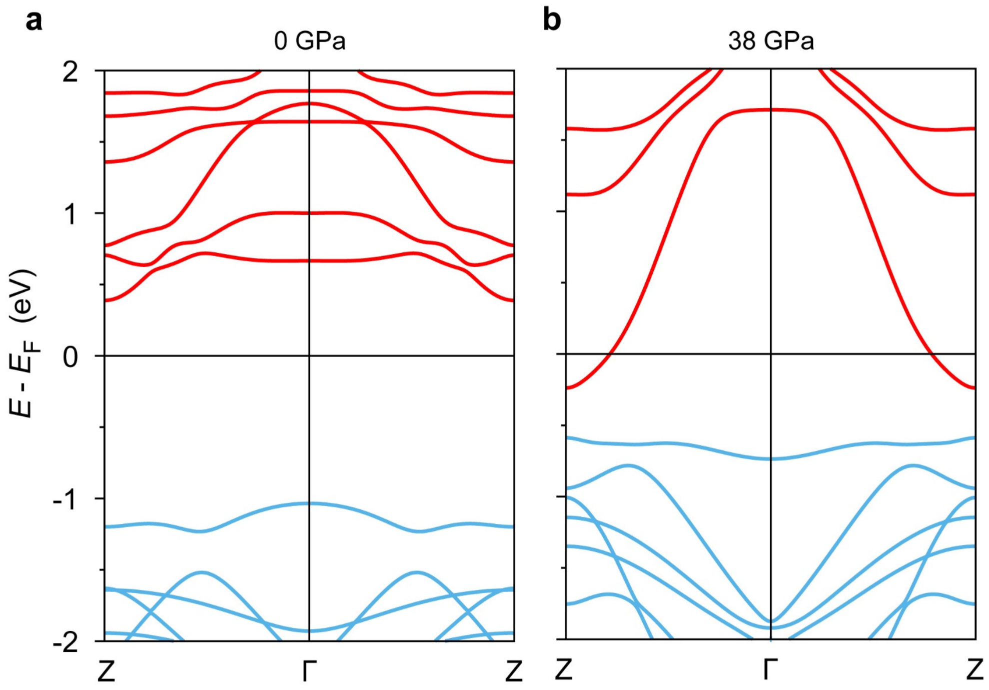

Supplementary Fig. 14 | Evolution of the electronic band structure of GeP. a–b, Calculated band structure (conduction bands in red and valence bands in blue) along the stacking direction Z–Γ–Z of the Brillouin zone at **a**, 0 GPa and **b**, 38.0 GPa.

To further understand how this can lead to the observed quasi-2D superconductivity, we also need to examine the coherence length of the Cooper pairs against the thickness of the host system.

Specifically, the superconductivity will be three-dimensional (3D) when the superconductor thickness is significantly larger than the out-of-plane coherence length, while the superconductivity will be 2D when the superconductor thickness is comparable to (or smaller than) the out-of-plane coherence length. Therefore, by reducing the sample thickness, we can achieve dimensionality crossover of the superconductivity from 3D to 2D.

Generally, the coherence length of superconductors represents the interferential distance of paired electrons, which indicates the spatial extension of the superconducting wavefunction and determines the dimensionality of superconductivity, as schematically shown in Supplementary Fig. 15a. When the thickness of the superconductor is larger than the out-of-plane coherence length, the superconducting electrons can move inside the sample without any confinement in the out-of-plane direction, and thus the superconductor exhibits the 3D character. When the thickness of the superconductor approaches a length scale comparable to the out-of-plane coherence length, the Cooper pairs move in a limited region strongly confined by the superconductor thickness, and thus achieving 2D superconductivity.

Such dimensionality behavior of the superconductivity can be well distinguished by the temperature-dependent upper critical field when the magnetic field is parallel to the sample surface, in which the $H_{c2}^{\parallel}(T)$ curve satisfies the linear relation for the 3D superconductivity case based on 3D anisotropic GL theory, while it follows the square root relation for the 2D superconductivity case based on 2D GL theory.

For bulk GeP sample 1 (with a thickness of hundreds of nanometers), whose thickness is significantly larger than the out-of-plane coherence length, the relationships between temperature and critical magnetic fields ($H_{c2}^{\perp}$ and $H_{c2}^{\parallel}$) both satisfy the linear relations based

on 3D anisotropic GL theory (Supplementary Fig. 15b), suggesting the 3D superconductivity in such a thick sample. Importantly, for our thin GeP sample 3 (with a thickness of 29 nm), the temperature-dependent $H_{c2}^{\perp}$ and $H_{c2}^{\parallel}$ at 45.0 GPa (Supplementary Fig. 15c) can be well fitted with the phenomenological GL expressions for 2D superconductivity, based on which an effective superconducting thickness of $d_{SC} = 23$ nm was determined. Note that this value is comparable to the thickness of GeP (29 nm), suggesting the achievement of quasi-2D superconductivity in the thin GeP sample.

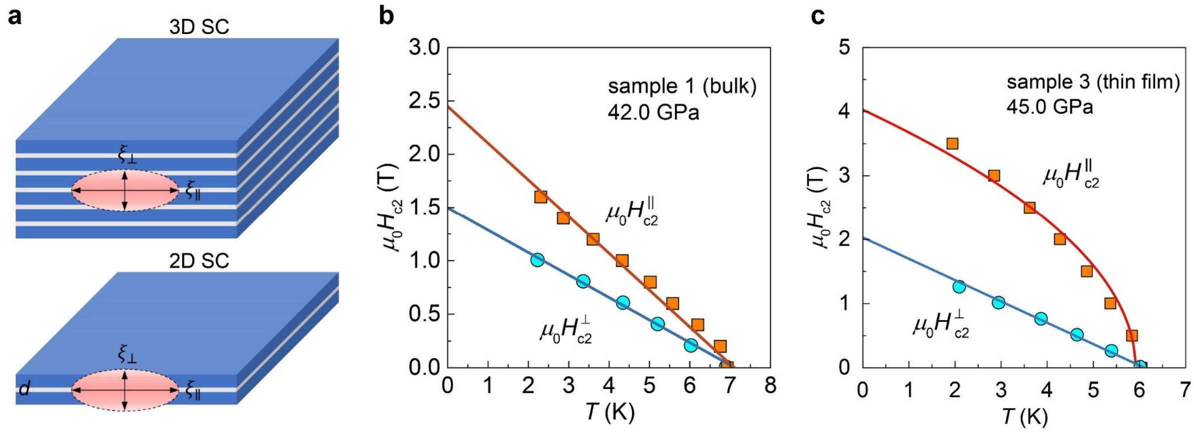

Supplementary Fig. 15 | Comparison of 2D and 3D superconductivity. a, Schematic illustration of the confinement effect of layered superconductors. In layered superconductors, the out-of-plane coherence length $\xi_\perp$ is smaller than the in-plane coherence length $\xi_\parallel$ in thick (top panel) and thin (bottom panel) samples. When the thickness of the superconductor is larger (smaller) than $\xi_\perp$, 3D (2D) superconductivity can be achieved. **b**, The $H_{c2}^{\parallel}$ and $H_{c2}^{\perp}$ of superconducting GeP (sample 1, bulk sample) at 42.0 GPa. Orange squares and blue dots represent $H_{c2}^{\parallel}$ and $H_{c2}^{\perp}$, and the fitted lines for both directions follow a linear relation based on the 3D anisotropic GL theory. **c**, The $H_{c2}^{\parallel}$ and $H_{c2}^{\perp}$ of superconducting GeP (sample 3, 29 nm) at 45.0 GPa. Orange squares and blue dots represent $H_{c2}^{\parallel}$ and $H_{c2}^{\perp}$. The fitted lines for the upper critical field of perpendicular and parallel cases follow the phenomenological 2D GL model.

8. Experimental confirmation of the quasi-2D nature of SC I and SC II superconducting phases

The angle-dependent upper critical field measurements are performed to further support the quasi-2D superconductivity of GeP (sample 3). The $H_{c2}(T)$ curves exhibit distinct temperature dependence for both $H_{c2}^{\parallel}$ and $H_{c2}^{\perp}$ under different pressures, as shown in Supplementary Fig. 16a, b. The $H_{c2}^{\parallel}(T)$ curves follow the typical behavior of 2D superconductors, which can be well fitted by equation (2) based on the 2D GL model. This is distinct from the linear behavior of the $H_{c2}^{\perp}(T)$ curves. We thus conclude that the present sample under pressure is in the quasi-2D superconducting regime.

The angular dependence of H_{c2} further supports this conclusion. The corresponding $H_{c2}(\theta)$ curves commonly exhibit strong anisotropy in two different directions (Supplementary Fig. 15c, d). One can clearly see in Supplementary Fig. 16c, d that the cusp-like peak at $\theta = 90°$ emerges for each pressure. We used the 2D Tinkham model[5] and 3D GL model[6] to fit the $H_{c2}(\theta)$ curve taken in the whole angle range. Note that the cusp-like peak in $H_{c2}(\theta)$ at $\theta = 90°$ can be fitted by the 2D Tinkham model, rather than the 3D GL model. Even though SC I and SC II have a slight difference in crystal structure, we can confirm the quasi-2D superconductivity in both cases.

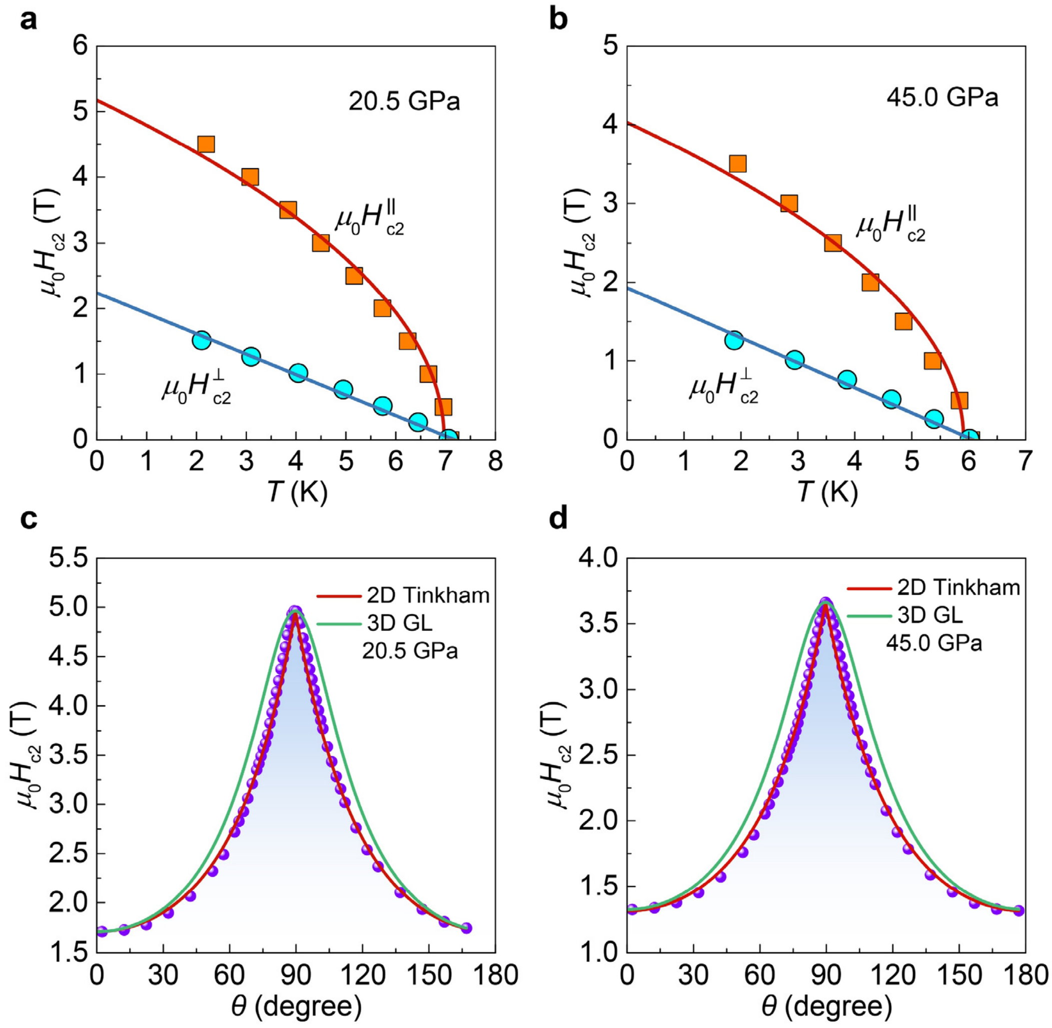

Supplementary Fig. 16 | Temperature-dependent H_{c2} curves and angle-dependent H_{c2} curves under different pressures. a–b, The $H_{c2}^{\parallel}$ and $H_{c2}^{\perp}$ of superconducting GeP (sample 3) at 20.5 GPa (**a**), 45.0 GPa (**b**). Orange squares and blue circles represent the $H_{c2}^{\parallel}$ and $H_{c2}^{\perp}$. The fitted lines for the upper critical field of perpendicular and parallel cases follow the phenomenological 2D GL model. **c–d**, Angular dependence of $H_{c2}(\theta)$ in sample 3 at 20.5 GPa (**c**), 45.0 GPa (**d**). The $H_{c2}(\theta)$ fitting with the 2D Tinkham model (red curve) and 3D GL model (green curve) clearly indicates that our experimental data can be well described with a 2D superconductivity scenario. The blue shaded areas in **c–d** correspond to the superconducting state.

9. $I(V)$ characteristics for BKT analysis under pressure

In order to understand the pressure dependence of the critical current in superconducting GeP, we carried out temperature-dependent current-voltage $I(V)$ measurements in the superconducting transition region of sample 1. The $I(V)$ characteristics as a function of temperature (Supplementary Fig. 17a, 17.2 GPa) changes from linear ohmic behavior to highly nonlinear behavior at low temperatures as the metal-superconductor transition occurs. The differential resistance $\mathrm{d}V/\mathrm{d}I$ is derived from the $I(V)$ characteristics of Supplementary Fig. 17a, and is plotted as a function of the current I in Supplementary Fig. 17b. With increasing temperature, the two broad symmetric peaks gradually become narrow and disappear around 9.0 K. The zero-resistance plateau for finite current at low temperature demonstrates the expected behavior for superconductivity. Supplementary Fig. 17c displays the critical current I_c as a function of temperature under different pressures, and I_c versus T can be fitted well with the empirical formula:

$$I_\mathrm{c}(T) = I_\mathrm{c}(0)\big(1 - (T/T_\mathrm{c})\big)^2 \qquad \mathrm{S}(1)$$

In the range of 17.2 to 25.0 GPa, the fitting parameter $I_\mathrm{c}(0)$ gradually increases with increasing pressure. Supplementary Fig. 17d shows the $I(V)$ characteristics at 2.0 K under different pressures. The pressure dependence of the critical current I_c was evaluated from Supplementary Fig. 17d using the value of the sharp peak ($\mathrm{d}V/\mathrm{d}I$) criteria, as shown in the inset in Supplementary Fig. 17d. The critical current increased monotonically with increasing pressure. At pressures up to 23.0 GPa, the critical current starts to saturate. This critical pressure is consistent with the pressure of the structural phase transition at P_M.

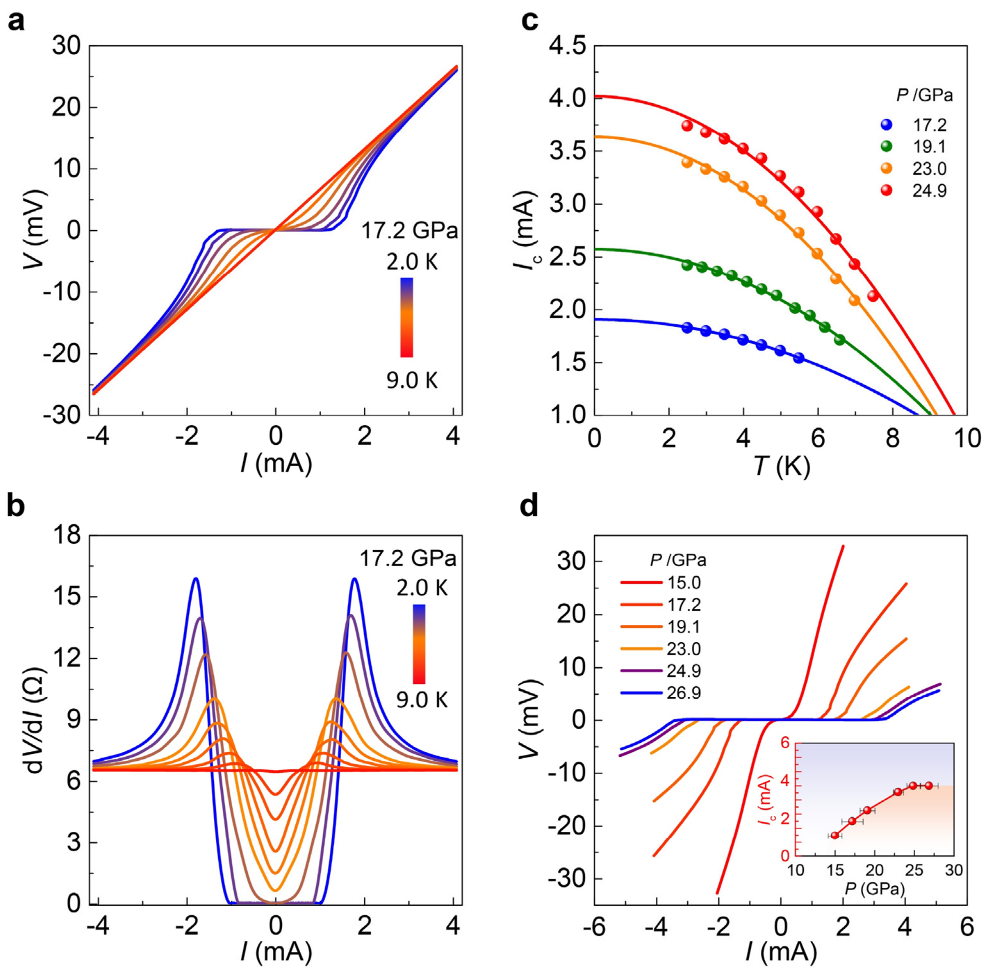

Supplementary Fig. 17 | $I(V)$ characteristics for BKT analysis under pressure. a, $I(V)$ characteristics at different temperatures close to T_c (ranging from 2.0 to 9.0 K) at 17.2 GPa. **b**, The differential resistance curves (ranging from 2.0 to 9.0 K), derived from the $I(V)$ relationship of (**a**). **c**, Temperature-dependent I_c for GeP under various pressures of 17.2, 19.1, 23.0 and 24.9 GPa. Solid lines represent the fits based on equation (S1). **d**, $I(V)$ characteristics under different pressures of 15.0, 17.2, 19.1, 23.0 and 24.9 GPa. The inset shows the critical current as a function of pressure, in which the metal phase is shaded in purple and the superconducting phase is shaded in red. The error bars on pressure are determined by the differences in fluorescence lines at different positions on the same ruby.

10. High-pressure differential conductance spectra of Andreev reflection

To understand the superconducting gap features of the pressurized GeP flakes, high-pressure differential conductance spectra $\mathrm{d}I/\mathrm{d}V$ of Andreev reflection were obtained based on a GeP/Graphite (GeP/G) heterostructure (sample 4) within the DAC. Previous reports on tunneling spectra have been successfully demonstrated at ambient pressure on $Bi_2Sr_2CaCu_2O_{8+\delta}$/Graphite heterostructure and ion-gated MoS_2 sandwich heterostructure[7,8]. A similar planar GeP/G heterostructure, shown in Supplementary Fig. 18a, is used as the device geometry in our differential conductance spectra measurements. Supplementary Fig. 18b, c displays the $R(T)$ and $R(H)$ curves of the GeP and GeP/G heterostructure at 24.0 GPa. The finite residual resistance induced by graphite as a background is involved in lifting the measured resistance of the GeP/G heterostructure.

Supplementary Fig. 18d shows the evolution of the normalized conductance curves with increasing temperature. One can see broad peaks in the differential conductance spectra, which can be well fitted by the Blonder-Tinkham-Klapwijk (BTK) model[9] (Fig. 4e), indicating that the obtained differential conductance spectra are dominant in the Andreev reflection regime. The broad peaks in the differential conductance spectra decrease with increasing temperature and eventually vanish at the superconducting critical temperature. The experimental data are well fitted with the BTK model, and the temperature dependence of the gap values obtained from the fitting is shown in Fig. 4f. Meanwhile, Supplementary Fig. 18e shows the magnetic field-dependent normalized conductance of the GeP/G heterostructure performed at 1.5 K and fit to the BTK model. The obtained gap values as a function of the magnetic field are thus shown in Supplementary Fig. 18f, which displays linear behavior. Such clear signatures of differential conductance spectra demonstrate that the energy-resolved spectroscopy within DAC is achieved, paving a new way for studying the differential conductance spectra for gap features and understanding the superconducting pairing mechanism under high pressure.

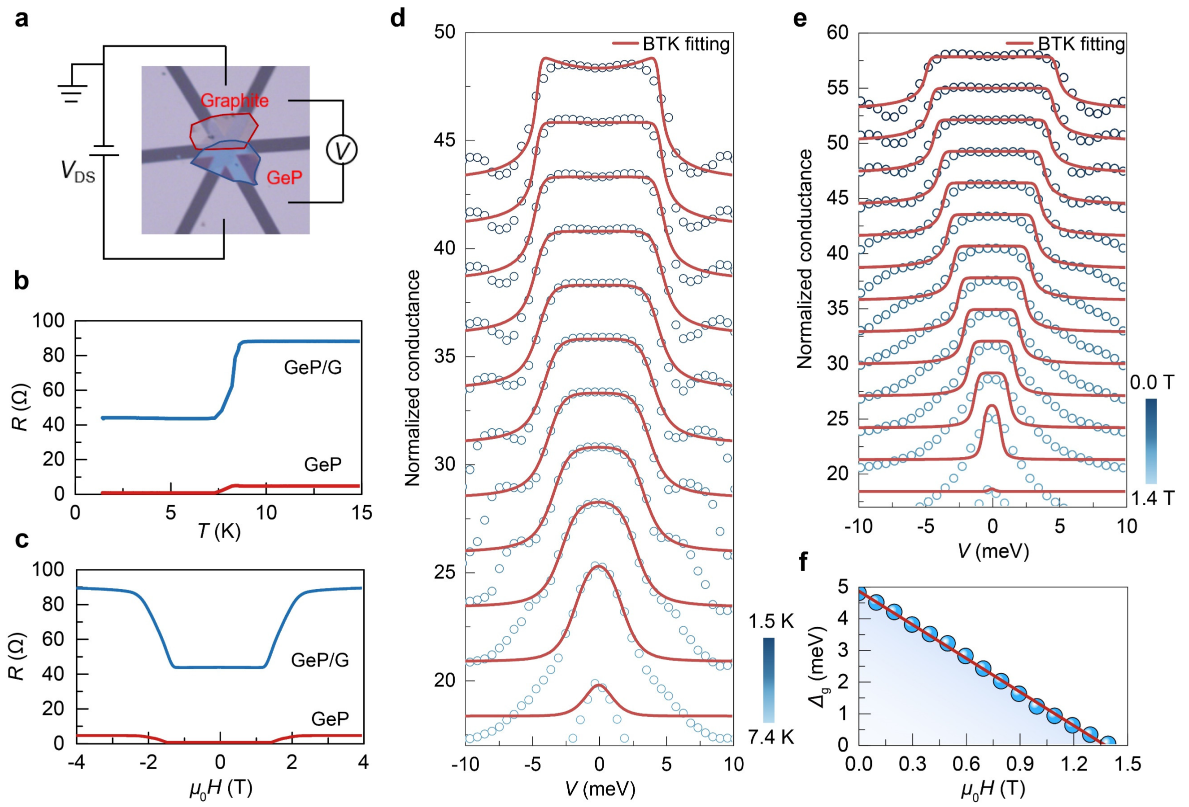

Supplementary Fig. 18 | High-pressure Andreev reflection of the GeP/G heterostructure. **a**, Optical image of the GeP/G heterostructure for the Andreev reflection, in which the measurement geometry is schematically shown. The graphite and GeP are highlighted by red and blue polygons. **b**, The $R(T)$ curves of GeP (red curve) and the GeP/G heterostructure (blue curve) at 24.0 GPa. **c**, The $R(H)$ curves of GeP (red curve) and the GeP/G heterostructure (blue curve) at 1.5 K at 24.0 GPa. **d**, Temperature dependence of the normalized differential conductance spectra ranging from 1.5 to 7.4 K. The curves are the fitting results of the BTK model. **e**, Magnetic field dependence of the normalized differential conductance spectra ranging from 0.0 to 1.4 T. The curves are the fitting results based on the BTK model. **f**, The obtained superconducting gap values as a function of the magnetic field (blue balls). The gap values can be fitted by a linear function (red line). The blue shaded area corresponds to the superconducting state.

References

1. Larson, A. C. & Von Dreele, R. B. General Structure Analysis System (GSAS). *Los Alamos National Laboratory Report LAUR*. 86–748 (2000).
2. Yang, F. et al. Ultrathin few-layer GeP nanosheets via lithiation-assisted chemical exfoliation and their application in sodium storage. *Adv. Energy Mater.* **10**, 1903826 (2020).
3. Yu, T. et al. Two-dimensional GeP-based broad-band optical switches and photodetectors. *Adv. Opt. Mater.* **8**, 1901490 (2019).
4. Zhao, S. et al. Flux method growth of large size group IV–V 2D GeP single crystals and photoresponse application. *Crystals* **11**, 2073–4352 (2021).
5. Tinkham, M. Effect of fluxoid quantization on transitions of superconducting films. *Phys. Rev.* **129**, 2413–2422 (1963).
6. Tinkham, M. *Introduction to Superconductivity* (Dover, NewYork, Ed. 2nd, 2004).
7. Liao, M. et al. Superconductor-insulator transitions in exfoliated $Bi_2Sr_2CaCu_2O_{8+\delta}$ flakes. *Nano Lett.* **18**, 5660–5665 (2018).
8. Costanzo, D., Zhang, H., Reddy, B. A., Berger, H. & Morpurgo, A. F. Tunnelling spectroscopy of gate-induced superconductivity in MoS_2. *Nat. Nanotechnol.* **13**, 483–488 (2018).
9. Blonder, G. E., Tinkham, M. & Klapwijk, T. M. Transition from metallic to tunneling regimes in superconducting microconstrictions: Excess current, charge imbalance, and supercurrent conversion. *Phys. Rev. B* **25**, 4515–4532 (1982).
